# Development of a Novel Immune Subtyping System Expanded with Immune Landscape and an 11-Gene Signature for Predicting Prostate Cancer Survival

**DOI:** 10.1155/2022/1183173

**Published:** 2022-02-16

**Authors:** Nan Li, Kai Yu, Zhong Lin, Dingyuan Zeng

**Affiliations:** ^1^Reproductive Medicine Center, Liuzhou Maternity and Child Health Care Hospital, Liuzhou 545001, China; ^2^Guangxi Health Commission Key Laboratory of Birth Cohort Study in Pregnant Women of Advanced Age, Liuzhou 545001, China; ^3^Affiliated Maternity Hospital and Affiliated Children's Hospital of Guangxi University of Science and Technology, Liuzhou 545001, China; ^4^College of Animal Science and Technology, Guangxi University, Nanning 530004, China; ^5^The Guangxi Zhuang Autonomous Region Reproducitve Hospital, Nanning 530021, China; ^6^The Department of Obstetrics and Gynecology, Liuzhou Maternity and Child Health Care Hospital, Liuzhou 545001, China

## Abstract

**Background:**

Nearly half of patients with prostate cancer will develop metastasis. Immunotherapy is currently a promising strategy for treating metastatic prostate cancer. This study aimed to construct an immune subtyping system and provide a more comprehensive understanding of tumor microenvironment.

**Methods:**

Data were downloaded from TCGA database and cBioPortal database. Consensus clustering was used to identify immune subtypes. Immune features were scored by ESTIMATE and CIBERSORT. Efficacy of different subtypes in immunotherapy was predicted by TIDE tool. Immune landscape was delineated through “monocle.” Coexpressed gene modules were identified by weighted correlation network analysis. Univariate Cox regression analysis and LASSO analysis were applied to construct a prognostic model.

**Results:**

Four immune subtypes (IS1 to IS4) were identified. Prognosis, mutation patterns, expression of immune genes, immune biomarkers, immunohistochemical biomarkers, and prediction efficacy of immunotherapy were significantly different among four immune subtypes. Five coexpressed gene modules were identified and an 11-gene prognostic model was constructed based on the modules.

**Conclusions:**

The study developed a novel immune subtyping system and an 11-gene prognostic model of prostate cancer, which could guide personalized treatment and immunotherapy for patients with prostate cancer.

## 1. Introduction 

Prostate cancer (PCa) is a commonly diagnosed male malignancy, which accounted for 10% of cancer-caused deaths [1]. Traditional therapies such as androgen depravation therapy (ADT), radiotherapy, and radical prostatectomy are usually applied treatment options for PCa patients, but not all patients will develop a positive prognosis. Over 40% of PCa patients with prostatectomy will experience disease recurrence [2]. ADT is the mainstay of managing metastatic PCa, despite an initial active response during the treatment, metastatic castration resistant prostate cancer (mCRPC) still occurs to a majority of patients [3].

Immunotherapy has been greatly improved in treating various cancers in the last decades, especially in the management of renal cell carcinoma, melanoma, and lung cancer [4–6]. Particularly, immune checkpoint inhibitors have been reported to possess impressive efficacy. However, only a small number of PCa show positive response to immunotherapy. Evidence proved that tumor microenvironment (TME) plays an essential role in each process of tumorigenesis, driving the outcome of prognosis [7]. The different components of TME can result in differential efficacy of immunotherapy. Although biomarkers have been widely explored to predict PCa prognosis [8–10], a guide tool for predicting the response of immunotherapy has not been developed.

In the present study, we aimed to comprehensively characterize an immune signature for PCa. We proposed a novel molecular subtyping system based on immune genes to predict the efficacy of immunotherapy. An immune landscape was delineated to complementary the immune subtyping system, and a prognostic model was constructed to predict the overall survival (OS) of PCa patients. Analysis was performed according to workflow diagram (Supplementary Figure S1).

## 2. Materials and Methods

### 2.1. Data Acquisition

TCGA-PRAD dataset including RNA-Seq and CNV data was downloaded from TCGA database (https://portal.gdc.cancer.gov/). MSKCC-PRAD dataset (MSKCC, Cancer Cell 2010) was downloaded from cBioPortal database (https://www.cbioportal.org/). TCGA-PRAD and MSKCC-PRAD were defined as training dataset and validation dataset, respectively. A total of 1989 immune-related genes including immune cell-specific genes, genes of costimulatory and coinhibitory molecules, genes of cytokines and cytokine receptors, genes for antigen processing and presentation, and other immune-related genes (Supplementary Table S1) were collected by extensively reviewing previous studies. In the below strategies, we followed the methods of Xia et al. [11].

### 2.2. Data Preprocessing

In TCGA-PRAD dataset, samples without survival data were excluded, while those whose transcripts per million (TPM) = 0 were in more than 50% samples were excluded. Ensembl gene ID was transferred to gene symbol. 495 samples and 20088 genes in TCGA-PRAD dataset were retained (Supplementary Table S2). In MSKCC-PRAD dataset, samples without survival data and probes without value were also excluded. Probes were matched to gene symbol, but one probe mapped to multiple genes was excluded. Median value of expression data was calculated if multiple probes mapped to one gene. Finally, 63 samples and 22486 genes were included in this study (Supplementary Table S3).

### 2.3. Identification of Immune Subtypes

ConsensusClusterPlus R package was performed to cluster immune-related genes from TCGA-PRAD dataset [12]. Partitioning around medoids (PAM) algorithm and Canberra distance was employed in consensus clustering. 80% of the total samples in TCGA-PRAD dataset were included in each time of bootstrap, which was implemented for 500 times. Groups (*k*) were set from 2 to 10, and the most optimized clusters were determined by cumulative distribution function (CDF) curve and consensus CDF. Kaplan–Meier survival curve and log-rank test were used to evaluate the performance of the immune subtyping system.

### 2.4. Immune Landscape of PCa

Monocle is an unsupervised algorithm and has been previously used to reduce dimensionality and construct a two-dimensional landscape [13]. The algorithm of Monocle represented the expression data of each sample as a point in a high-dimensional Euclidean space, allowing each sample to be casted as a point in the two-dimensional graph. Finally, a tree structure manifesting the immune features of each sample was established by Monocle.

### 2.5. Identification of Coexpressed Gene Modules

Weighted correlation network analysis (WGCNA) R package was performed to identify immune-related gene modules [14]. The most optimized cluster was defined with a condition of the negative relation between log(*k*) and log(*p*(*k*)), *R*^2^ > 0.85, and soft threshold (power) = 12. Topological overlap matrix (TOM) was established based on adjacency matrix. We applied average-linkage hierarchical clustering and dynamic branch cutting to identify co-expression modules that contained at least 30 genes.

### 2.6. Gene Enrichment Analysis

Single sample gene set enrichment analysis (ssGSEA) in the GSVA R package was implemented to score immune cells [15]. ANOVA was performed to assess the relation between immune subtypes and 56 types of immune-related biomarkers [16]. Enriched biological processes in gene ontology (GO) terms of six immune-related gene modules were annotated by David (v6.8) [17].

### 2.7. Identification of Prognostic Model

Univariate Cox regression analysis was conducted to identify gene modules and prognostic genes significantly correlated with OS in TCGA-PRAD dataset. Least absolute shrinkage and selection operator (LASSO) regression in the glmnet R package and stepAIC in the MASS R package were applied to reduce the quantity of prognostic genes and optimize the prognostic model [18, 19]. Risk score was defined as coefficient 1*∗*gene 1 expression + coefficient 2*∗*gene 2 expression + ⋯ + coefficient n*∗*gene n expression. Kaplan–Meier survival curve and log-rank test were used to evaluate the model performance.

## 3. Results

### 3.1. Construction and Validation of Immune Subtypes of Prostate Cancer

**Figure 1:**
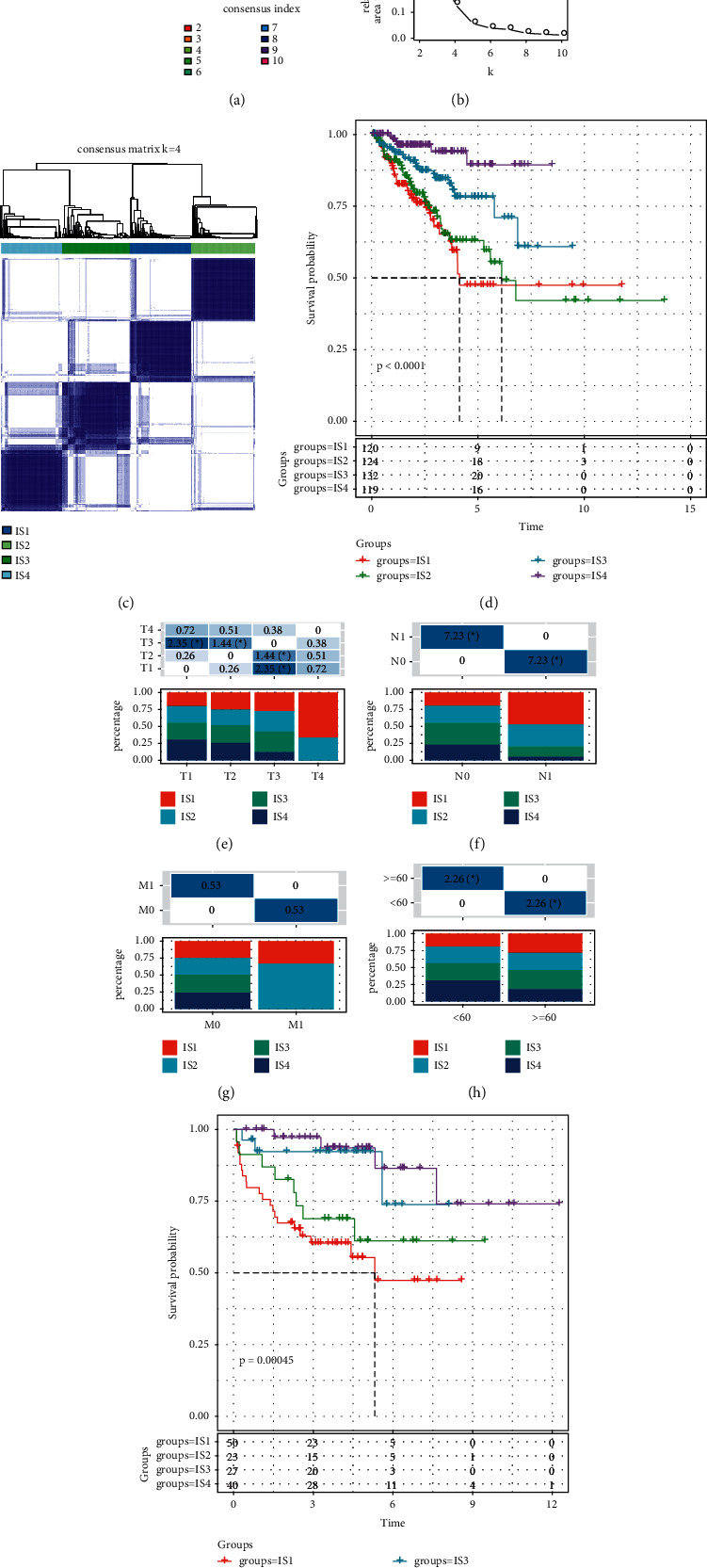
Four immune subtypes of PCa and its relation with clinical features. (a) CDF curve containing cluster numbers *k* from 2 to 10. (b) CDF delta area curve with *k* = 2 to 10. (c) The consensus matrix when *k* = 4. (d) Kaplan–Meier survival curve of four immune subtypes within TCGA-PRAD dataset. (e-g) The distribution of four immune subtypes in clinical features including T stage (e), N stage (f), M stage (g), and age (h). ANOVA was performed. (i) Kaplan–Meier survival curve of four immune subtypes within MKSCC-PRAD dataset. Log-rank test was performed ^*∗*^*p* < 0.05.

Gene expression profiles of 1909 immune-related genes in TCGA-PRAD dataset were extracted initially. After conducting univariate Cox regression analysis, a total of 534 immune-related genes were found to be significantly associated with OS. Gene expression profiles of these 534 genes were then used to determine molecular subtypes. According to the algorithm of consensus clustering, the optimal cluster was defined by cluster numbers (*k*) from 2 to 10. The most stable cluster when *k* = 4 (Figures 1(a) and 1(b)) was delineated by CDF and CDF delta area curves, and four immune subtypes (IS, IS1 to IS4) were constructed (Figure 1(c)). Survival analysis revealed that the four immune subtypes varied in OS; specifically, IS4 group had the favorable prognosis, while IS1 group had the worst prognosis (*p* < 0.0001, Figure 1(d)). Moreover, we also described the distribution of four immune subtypes in the conventional TNM staging system within TCGA-PRAD dataset. The analysis showed that the proportion of IS4 group decreased from T1 to T4, N0 to N1, and M0 to M1, while the proportion of IS1 group increased oppositely, which was consistent with the tendency of disease progression (Figures 1(e)–1(g)). In addition, a significant difference of distribution of immune subtypes was also observed between age <60 and age ≥60 groups (Figure 1(h)). To further verify the robustness of this immune subtyping system, another independent dataset, MKSCC-PRAD dataset, was classified into four groups. Similarly, significant difference was shown within four immune subtypes, and IS4 group still showed the best OS (Figure 1(i)).

### 3.2. Tumor Mutation Burden and Mutation Patterns of Four Immune Subtypes

**Figure 2:**
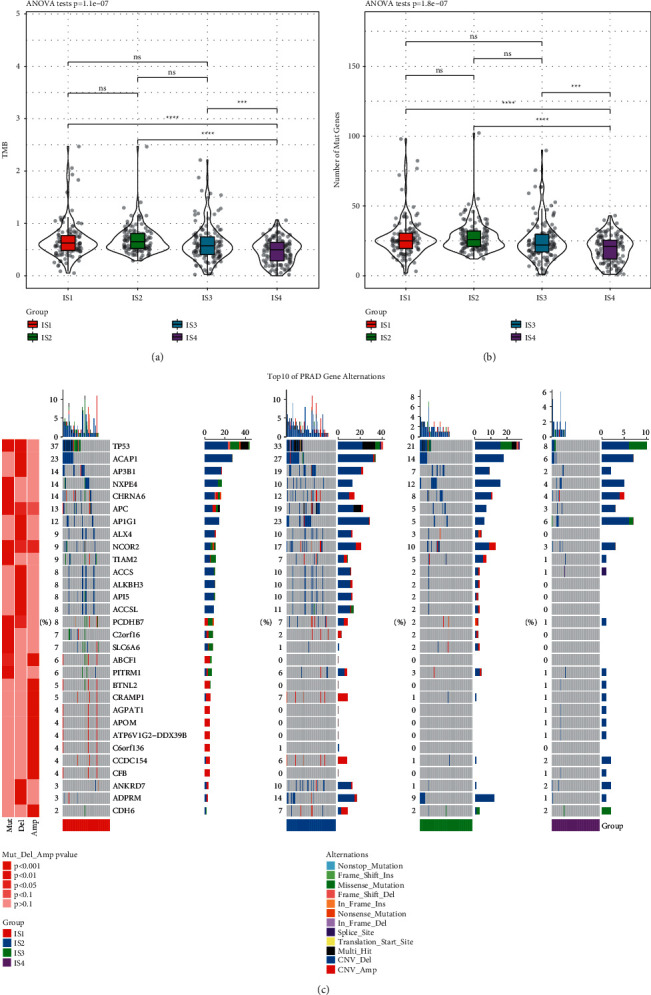
Mutation analysis in TCGA-PRAD dataset. The distribution of TMB (a) and number of mutated genes (b) in four immune subtypes. ANOVA was performed. (c) Mutation patterns and 12 types of variations in four groups. Fisher's exact test was used ^∗∗∗^*p* < 0.001, ^∗∗∗∗^*p* < 0.001.

In TCGA-PRAD dataset, mutect2 software was employed to calculate tumor mutation burden (TMB). IS4 group showed the lowest TMB and number of mutated genes when compared with other groups (*p* < 0.001, Figures 2(a) and 2(b)). We further assessed the mutation patterns of each group. Copy number alternations, especially deletions, were the majority mutations in all groups (Figure 2(c)). Reasonably, IS1 group comprised the largest amount of mutations, and IS4 group had the least mutations. The top 10 mutated genes were TP53, ACAP1, AP3B1, NXPE4, CHRNA6, APC, AP1G1, ALX4, NCOR2, and TIAM2. The mutation frequencies of TP53, NXPE4, and CHRNA6 were the highest in IS1 group, while ACAP1, AP3B1, APC, AP1G, 1NCOR2, and ADPRM genes showed the most mutations in IS2 group (*p* < 0.001). Interestingly, the frequencies of copy number variations of BTNL2, AGPAT1, APOM, ATP6V1G2-DDX39B, C6orf136, CCDC154, and CFB genes were greatly higher than other groups (*p* < 0.001).

### 3.3. Differential Expression of Chemokines, Chemokine Receptors, and Immune Checkpoints among Four Immune Subtypes

**Figure 3:**
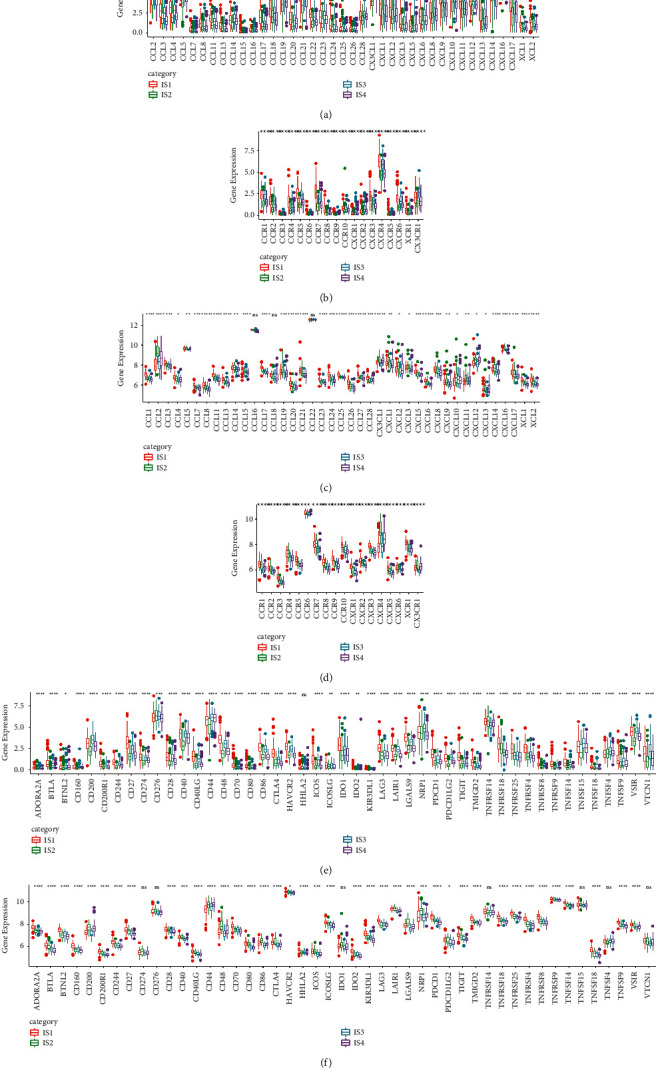
The differential expression of chemokines, chemokine receptors, and immune checkpoints among four immune subtypes. (a-b) The expression of chemokines (a) and chemokine receptors (b) in TCGA-PRAD dataset. (c-d) The expression of chemokines (c) and chemokine receptors (d) in MKSCC-PRAD dataset. (e-f) The expression of total 47 immune checkpoints in TCGA-PRAD dataset (e) and MKSCC-PRAD dataset (f). ANOVA was performed. ^*∗*^*p* < 0.05, ^∗∗^*p* < 0.01, and ^∗∗∗^*p* < 0.001. ns: no significance.

Chemokines together with cytokines play a critical role in TME. Chemokine receptors secreted by tumor cells are involved in tumor proliferation and metastasis and can serve as biomarkers of immunotherapy. Therefore, we evaluated the expression of chemokines and chemokine receptors and compared in the four immune subtypes. In TCGA-PRAD dataset, a total of 39 types of chemokines were expressed; noticeably, the expression level of each gene varied significantly among four immune subtypes (*p* < 0.01, Figure 3(a)), and the expression of chemokine receptors was also differential among the four groups (*p* < 0.01, Figure 3(b)). In MKSCC-PRAD dataset, 38 out of 41 chemokines expressed differentially, and the expression of chemokine receptors was differential among the four groups (*p* < 0.05, Figures 3(c) and 3(d)). Furthermore, the expression level of immune checkpoints was calculated. Among 47 immune checkpoints, 46 genes expressed differentially in TCGA-PRAD dataset, and 40 genes expressed differentially in MKSCC-PRAD dataset (*p* < 0.05, Figures 3(e) and 3(f)). These results supported the fact that the expression of chemokines, chemokine receptors, and immune checkpoints was different among IS1, IS2, IS3, and IS4 groups.

### 3.4. Differential Expression of PCa Immunohistochemical Biomarkers

**Figure 4:**
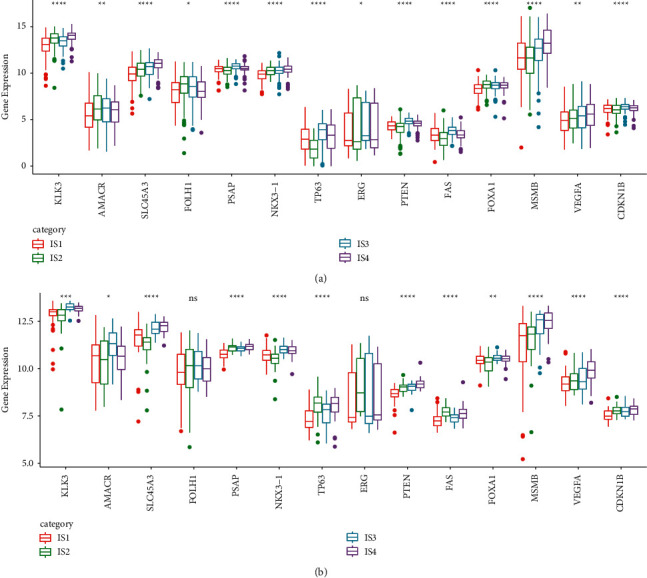
The differential expression of PCa immunohistochemical biomarkers in TCGA-PRAD dataset (a) and MKSCC-PRAD dataset (b). ANOVA was performed. ^*∗*^*p* < 0.05, ^∗∗^*p* < 0.01, ^∗∗∗^*p* < 0.001, and ^∗∗∗∗^*p* < 0.0001. ns: no significance.

Immunohistochemistry is commonly used in biopsy, and prostate-specific antigen (PSA) is one of the most popularly performed tests in PCa. To examine whether there was a correlation between immune subtypes and PCa immunohistochemical biomarkers, we incorporated a series of biomarkers currently used from Abcam website (https://www.abcam.cn/cancer/). In both TCGA-PRAD and MKSCC-PRAD datasets, significant expression difference of biomarkers among IS1, IS2, IS3, and IS4 groups was detected. There was no difference of FOLH1 and ERG in MKSCC-PRAD dataset, but the remaining biomarkers were all differentially expressed among the four groups (Figure 4).

### 3.5. Immune Features of Four Immune Subtypes

**Figure 5:**
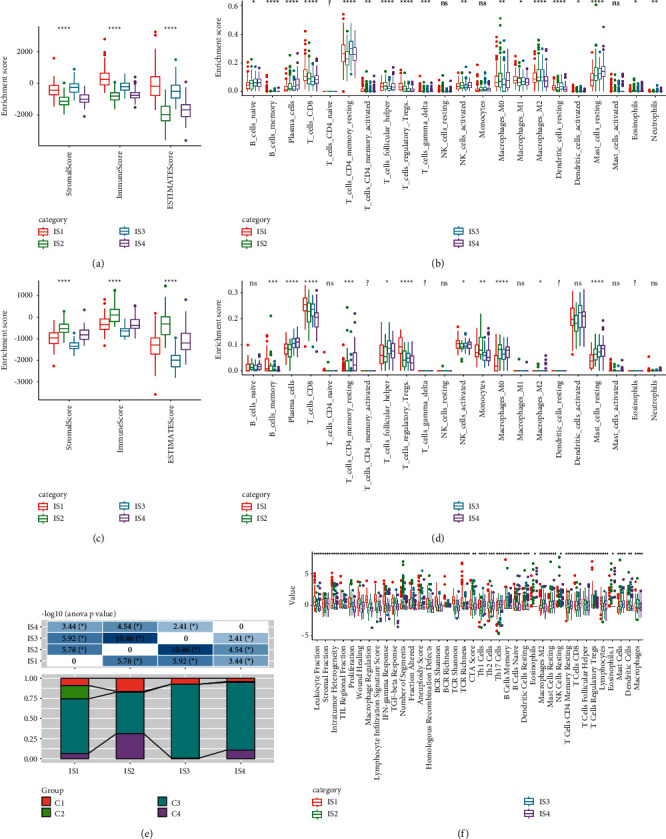
Differential immune features of immune subtypes. (a-b) Immune features of four immune subtypes scored by ESTIMATE (a) and CIBERSORT (b) tools in TCGA-PRAD dataset. (c-d) Immune features of four immune subtypes scored by ESTIMATE (c) and CIBERSORT (d) tools in MKSCC-PRAD dataset. (e) The distribution of C1 to C4 groups in IS1 to IS4 groups. (f) 38 immune biomarkers significantly varied in IS1 to IS4 groups. ANOVA was performed. ^*∗*^*p* < 0.05, ^∗∗^*p* < 0.01, ^∗∗∗^*p* < 0.001, and ^∗∗∗∗^*p* < 0.0001. ns: no significance. ^?^Low expression cannot be calculated.

To investigate whether there was immune heterogeneity among the four immune subtypes, ESTIMATE and CIBERSORT tools were applied to score the samples in TCGA-PRAD and MKSCC-PRAD datasets. The enrichment score of the two datasets significantly varied among the four immune subtypes (Figures 5(a)–5(d)). In TCGA-PRAD dataset, IS1 group had the highest ESTIMATE score, but IS2 group had the lowest ESTIMATE score (*p* < 0.0001, Figure 5(a)). In MKSCC-PRAD dataset, IS2 group had the highest ESTIMATE score, but IS3 group had the lowest ESTIMATE score (*p* < 0.0001, Figure 5(c)). 22 types of immune cells were scored by CIBERSORT tool. In the two datasets, IS4 group exhibited a high enrichment score in plasma cells, macrophages M0, and resting mast cells, while IS1 group showed a high score in CD8+ T cells and regulatory T cells (*p* < 0.0001, Figures 5(b) and 5(d)).

A pan-cancer research classified cancers into six immune subtypes C1 to C6 based on IFN-*γ*, TGF-*β*, macrophage, lymphocyte, and wound healing, and PCa was stratified into C1 to C4 four groups [16]. Reasoning that the same TCGA-PRAD dataset was used, a comparison between C1 to C4 groups and IS1 to IS4 groups was conducted in this study. A significant difference of C1 to C4 distribution was observed from IS1 to IS4 groups. C2 group mostly accumulated in IS1 group, and a majority of C1 and C4 groups were in IS2 group (*p* < 0.05, Figure 5(e)). Moreover, we evaluated the correlation between IS1 to IS4 groups and immune biomarkers from the literature [16]. A total of 56 immune biomarkers were included, and 38 of them had differential enrichment score among the four immune subtypes (FDR < 0.01, *p* < 0.05, Figure 5(f)). A majority of immune biomarkers were enriched in IS1 group, especially leukocyte fraction, macrophage regulation, lymphocyte infiltration, IFN-*γ* response, TCR Shannon, TCR richness, dendritic cells and lymphocytes; however, these biomarker were less enriched in IS2 group (*p* < 0.01, Figure 5(f)).

### 3.6. The Differential Performance of Immunotherapy within Four Immune Subtypes

**Figure 6:**
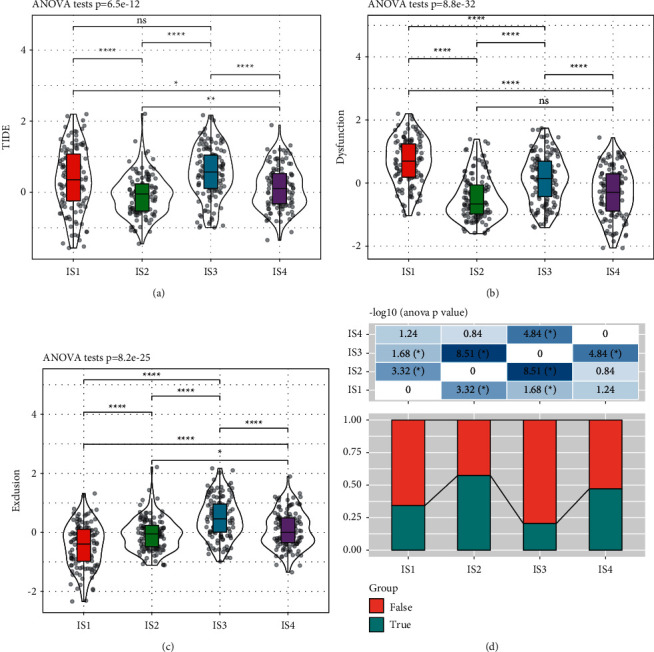
Prediction of immunotherapeutic efficacy among four immune subtypes. (a) Immune response scored by TIDE. (b-c) The performance of T cell dysfunction (b) and T cell exclusion (c) in four groups. (d) Prediction of immunotherapeutic efficacy in four groups. True and false represents the positive and negative immune response to immunotherapy, respectively. ANOVA was performed. ^*∗*^*p* < 0.05, ^∗∗^*p* < 0.01, ^∗∗∗^*p* < 0.001, and ^∗∗∗∗^*p* < 0.0001. ns: no significance.

We then analyzed the immunotherapeutic performance of IS1 to IS4 using TIDE software (http://tide.dfci.harvard.edu/). A higher TIDE score represents higher possibility of immune escape, indicating less benefit from immunotherapy. IS1 and IS3 groups showed higher TIDE score than IS2 and IS4 groups, indicating lower effectiveness of immunotherapy of IS1 and IS3 groups (*p*=6.5*e* − 12, Figure 6(a)). In addition, we also calculated the scores of T cell dysfunction and T cell exclusion, as shown in Figures 6(b) and 6(c), respectively. T cell dysfunction was the strongest in IS1 group, and this was correlated with unfavorable survival, although its T cell exclusion score was the lowest. Immune response was significantly different among these immune subtypes, showing the worst immunotherapeutic efficacy in IS3 group and the optimal immune response in IS2 group (Figure 6(d)).

### 3.7. An Immune Landscape of PCa and an Extension for Immune Subtyping System

**Figure 7:**
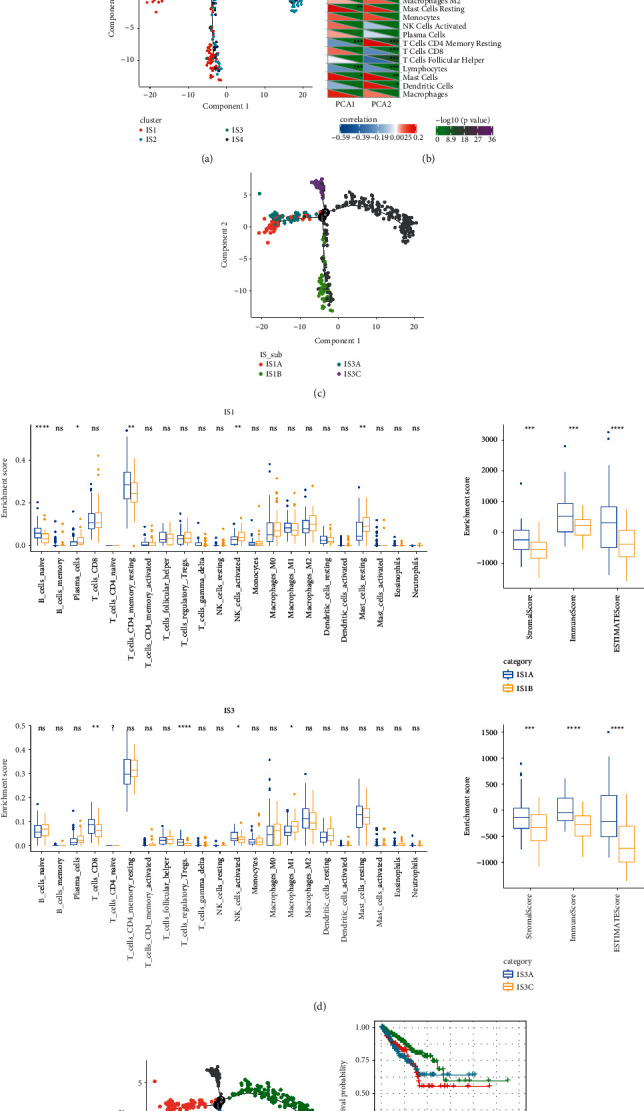
Construction of immune landscape within TCGA-PRAD dataset. (a) The distribution of four immune subtypes in the immune landscape. (b) The relation between component 1 (PCA1), component 2 (PCA2), and immune biomarkers. (c) Subdivision of IS1 and IS3 groups. (d) Immune features of IS1A and IS1B, IS3A, and IS3B scored by ESTIMATE and CIBERSORT tools. (e) Immune landscape grouped by branches 1, 3, and 5. (f) Kaplan–Meier survival curve of groups 1, 3, and 5. Log-rank test was performed. ^*∗*^*p* < 0.05, ^∗∗^*p* < 0.01, ^∗∗∗^*p* < 0.001, and ^∗∗∗∗^*p* < 0.0001. ns: no significance.

To further examine the immune features and subtypes of PCa, we applied a reduced dimensional method where each sample was casted as a point in a two-dimensional space in a latent tree structure. Component 1 and component 2 were two independent immune-related gene sets generated by principle component analysis. An immune landscape of PCa was constructed, and four immune subtypes were labeled with different colors (Figure 7(a)). Next, we assessed the correlation between two components and immune biomarkers. Component 1 was found to be negatively related to leukocyte fraction, macrophage regulation, lymphocyte infiltration signature score, TGF-*β* response, TCR Shannon, and TCR richness, which was consistent with the previous result (|*R*| > 0.5, *p* < 0.001, Figures 7(b) and 5(f)). Component 2 was significantly associated with wound healing, T cells follicular helper, IFN-gamma response, and T cells CD4 memory resting (*p* < 0.001, Figure 7(c)). According to the immune landscape, IS1 and IS3 groups could be further subdivided into IS1A and IS1B, IS3A and IS3B. The immune features of subgroups showed subtle difference between two groups scored by CIBERSORT, and differential enrichment score was calculated by ESTIMATE (Figure 7(d)). Additionally, survival analysis revealed that three branches of the tree structure showed differences in OS (*p*=0.036), indicating that this immune landscape was reliable and effective in further supplementary the immune subtypes (Figures 7(e) and 7(f)).

### 3.8. Identification of Coexpressed Gene Modules Based on Immune-Related Genes

**Figure 8:**
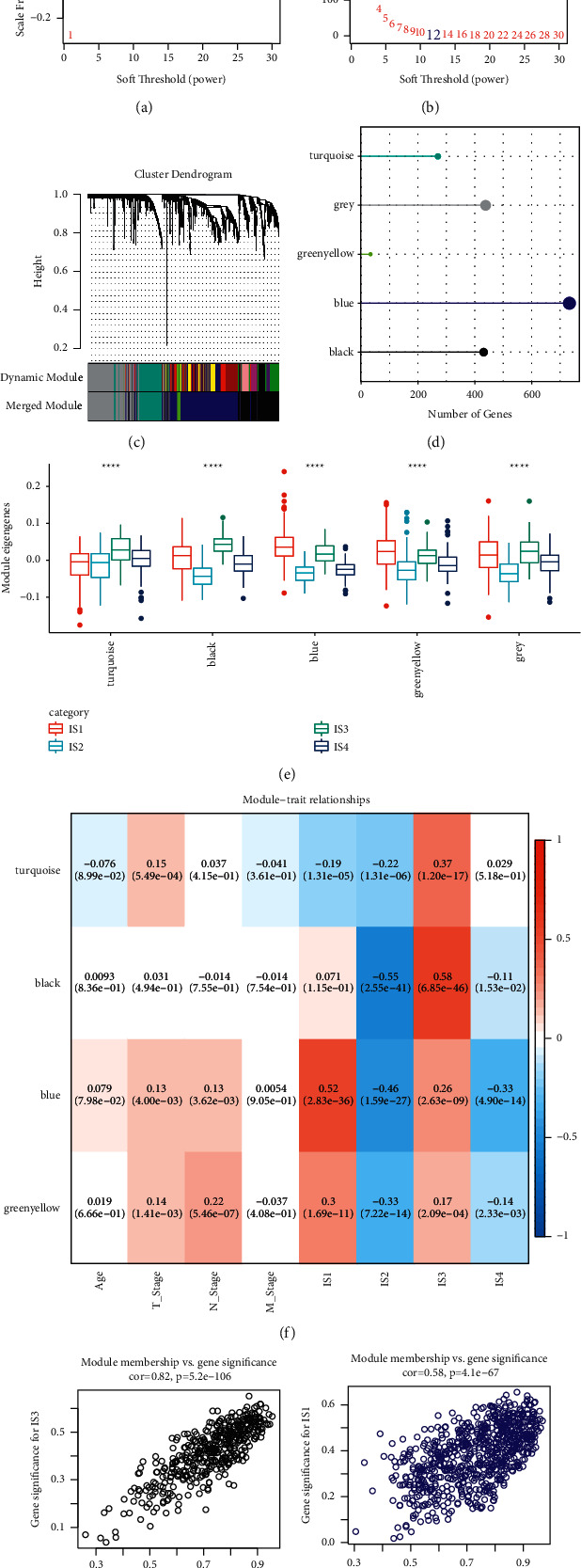
Identification of coexpressed gene modules. (a-b) Analysis of the scale-free fit index (a) and mean connectivity (b) for various soft-thresholding powers. (c) Cluster dendrogram and merged modules when soft-thresholding powers = 12. (d) Five modules colored with turquoise, grey, green-yellow, blue, and black. (e) Eigengenes of four immune subtypes groups by modules. (f) The correlation between modules and clinical features, immune subtypes. Positive correlation and negative correlation were colored with red and blue, respectively. (g-h) Scatter diagram of the relation between black module and IS3 group (g), blue module and IS1 group (h).^∗∗∗∗^*p* < 0.0001.

We also identified coexpressed gene modules to further explore immune-related genes by WGCNA. Under the condition of the negative relation between log(*k*) and log(*p*(*k*)), *R*^2^ > 0.85 and soft threshold (power) = 12 were defined to meet a scale-free network (Figures 8(a) and 8(b)). Using average-linkage hierarchical clustering and dynamic branch cutting, co-expression modules containing at least 30 genes in each module were identified. Modules with close distance were then merged, and five modules were identified when height = 0.3, deepSplit = 4, and minModuleSize = 30 (Figure 8(c)). Finally, 1905 immune-related genes were classified into five modules colored as turquoise, grey, green-yellow, blue, and black (Figure 8(d), Supplementary Table S4). In each module, all eigengenes varied significantly within four immune subtypes, which supported the effectiveness of the immune subtyping system (*p* < 0.0001, Figure 8(e)). Furthermore, close relation between modules and immune subtypes was demonstrated. IS1 and IS3 groups were positively related to modules, especially to the black and blue modules, while IS2 and IS4 were negatively correlated with the modules (Figure 8(f)). However, clinical features including age, T, N, and M stages were not tightly associated with modules. The scatter diagram demonstrated close association of black module with IS3 group (coefficient = 0.82, *p* < 0.0001) and blue module with IS1 group (coefficient = 0.58, *p* < 0.0001) (Figures 8(g) and 8(h)).

### 3.9. Function of Coexpressed Gene Modules and Screening of Prognostic Genes

**Figure 9:**
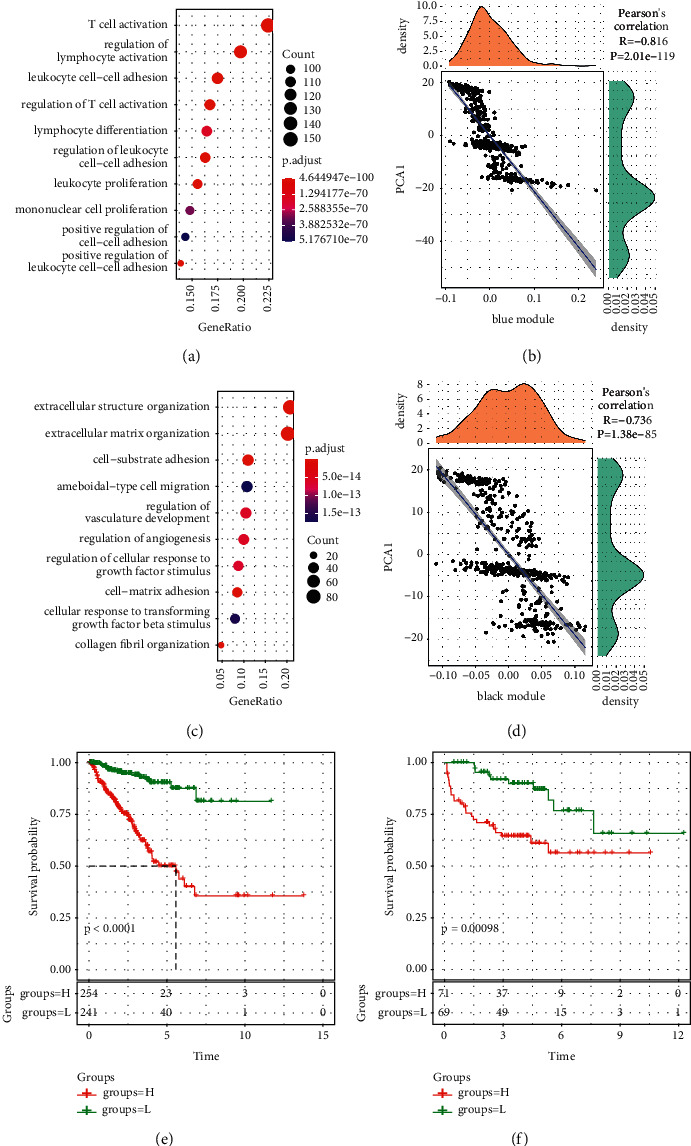
Functional analysis of blue and black modules, and survival analysis of two datasets. (a) Top 10 enriched biological processes in blue module. (b) Pearson's correlation between blue module and component 1. (c) Top 10 enriched biological processes in black module. (d) Pearson's correlation between black module and component 1. (e-f) Kaplan–Meier survival curve in TCGA-PRAD (e) and MKSCC-PRAD (f) datasets. H: high risk. L: low risk. Log-rank test was performed.

Gene set enrichment analysis was conducted to determine enriched biological processes of blue and black modules. The results showed that blue module was largely enriched to biological processes such as T cell activation, regulation of lymphocyte activation, and leukocyte proliferation, and it was negatively correlated with component 1 (*R* = −0.816, *p* < 0.0001, Figures 9(a) and 9(b)). For black module, biological processes of extracellular structure organization and extracellular matrix organization were enriched, and the module was also negatively correlated with component 1 (*R* = −0.736, *p* < 0.0001, Figures 9(c) and 9(d)).

Genes closely related to prognosis were screened, and a total of 243 genes with *R* > 0.85 were detected from the blue and black modules. LASSO regression analysis was applied to construct a prognostic model. When lambda = 016636511, the model was optimal, and 17 genes were identified. To further simply the model, we conducted Akaike information criterion to reach a high fitting degree through including the minimum amount of genes. Finally, based on FGD2, IL2RG, LRMP, NCF1, VAV1, ZNF831, COL5A1, EBF1, PCDH18, PLXND1, and PTGIS, an 11-gene prognostic model was defined as follows.

(1) $$\begin{array}{c} \text{Risk Score}=0.4463861*\text{FGD}2-0.3572187*\text{IL}2\text{RG}-0.5703754\\ {}*\text{LRMP}\mathrm{*0}\mathrm{.5567643*}\text{NCF}1+0.5364159*\text{VAV}1-0.3522158*\\ \text{ZNF}831+0.6454266*\text{COLA}1-0.4826737*\text{EBF}1-0.5881331*\\ \text{PCDH}18+0.4988597*\text{PLXNDI}-0.4001723*\text{PTGIS.} \end{array}$$

The risk score of each sample in TCGA-PRAD and MKSCC-PRAD datasets was calculated and converted to *z*-score, which was then used to divide the samples that were divided into high-risk or low-risk group. The result showed that OS in low-risk group was higher than high-risk group in both datasets (*p* < 0.001, Figures 9(e) and 9(f)). In addition, we compared the expression differences of these 11 genes in cancer and adjacent samples and observed that FGD2, LRMP, VAV1, EBF1, PCDH18, and PTGIS were significantly underexpressed in tumor samples (Supplementary Figure S2A). Further, we analyzed the relationship between these 11 genes and immune infiltration and observed that these genes were significantly related to multiple immune infiltrating cells, especially with T_ cells_ CD4_ memory_ Resting and dendritic_ cells_ Resting showed a significant positive correlation (Supplementary Figure S2B). The correlation analysis of immune checkpoint genes showed that ZNF831, VAV1, NCF1, LRMP, IL2RG, and FGD2 showed a significant positive correlation with a variety of immune checkpoint genes (Supplementary Figure S2C). Further, we mapped these 11 genes to the string database to analyze the interaction between these genes. It can be observed that there is little direct interaction between these genes, but more indirect interaction, suggesting that these genes may play different roles in different time and space (Supplementary Figure S2D).

## 4. Discussion

For mCRPC patients, immunotherapy is now the only available treatment. Sipuleucel-T, which is the only cancer vaccine approved by Food and Drug Administration (FDA) in treating mCRPC, was a significant improvement in mCRPC treatment [20, 21]. Extended OS was observed in the sipuleucel-T trials with tolerated adverse effects [21, 22]. According to a large-scale research on mCRPC patients, only approximately 10% could benefit from sipuleucel-T, indicating the limitation of the cancer vaccine in wide application [23]. Immune checkpoint inhibitors against PD-1, PD-L1, and CTLA-4 have found to be able to prolong the OS of mCRPC patients. However, the efficacy of current monoclonal antibodies is not satisfactory, and new clinical trials of updated strategies are still ongoing [24]. To some extent, immunotherapy of PCa is still far from mature. Evidence revealed that TME is of great importance for tumor progression and can suppress or stimulate the efficacy of immunotherapy [25]. Therefore, a comprehensive understanding of the TME of Pca plays a critical role in guiding immunotherapy.

In the current study, we explored an immune subtyping system that has not been reported before. Based on immune-related gene expression profiles of TCGA-PRAD dataset, a unique molecular subtyping system was generated through substantial informatics analysis. All patients could be classified into four immune subtypes (IS1 to IS4). The OS was different among the groups, with the optimal prognosis in IS4 group and the worst prognosis in IS1 group. The proportion of IS1 group in the TNM staging system was consistent with the progressing stages. In addition, IS1 group had the highest mutation frequency, especially increased copy numbers. The different mutation patterns may explain the differential component of TME.

Immune infiltration is a pivotal component of TME and represents the immune signatures of cancers. Chemokines are a family of chemotactic cytokines that can regulate the positioning and expression of immune cells [26, 27]. As chemokines and chemokine receptors are responsible for cancer metastasis, they have also been considered to be the possible targets of cancer immunotherapy [28]. The expression of chemokines and chemokine receptors in PCa was evaluated in our study. Differential expression was observed among the four immune subtypes, indicating that expression patterns of chemokines and chemokine receptors may result in different outcomes of PCa development.

According to the previous researches, tumors can be divided into three infiltration patterns (immune-inflamed or immune-active (‘hot'), immune-excluded, and immune-deserted (‘cold')) in terms of the components of TME [29, 30]. PCa has been stratified into immune-desert pattern and is inactively responsive to immunotherapy [31]; therefore, only a small number of patients can benefit from the immunotherapy. To ascertain whether the infiltration pattern would be different among the four immune subtypes, we assessed 56 immune biomarkers and scored their enrichment. The expression of immune biomarkers varied in four immune subtypes. The results showed that according to the enrichment level of leukocyte fraction, stromal fraction, TIL regional fraction proliferation, macrophage regulation, IFN-*γ* response, TCR richness, CD8+ T cells, and TGF-*β* response, IS1 group was classified into immune-excluded pattern and IS2 group was immune-desert pattern. Furthermore, TIDE analysis also revealed that patients in IS2 and IS4 groups were more suitable to receive immunotherapy than those in IS1 and IS3 groups. The specific stratification of infiltration patterns and efficacy prediction of immunotherapy can provide a guidance for personalized immunotherapy.

By introducing a graph-learning landscape, IS1 and IS3 groups were further subdivided. The enrichment of immune biomarkers was significantly different in the subdivisions. The immune landscape of PCa supplemented the immune subtyping system and visualized the immune signatures, providing a better understanding of the tumor microenvironment. In addition, co-expression gene modules were constructed, and 11 prognostic genes were identified from the models. The 11-gene prognostic model can predict the prognosis and further facilitate personalized treatment of PCa.

## 5. Conclusion

In conclusion, we defined a new molecular subtyping system based on immune-related genes. PCa patients were classified into four immune subtypes and showed significant difference in prognosis, immune signatures, response of immunotherapy, and infiltration patterns. An immune landscape of PCa was generated and helps further understand the TME. This novel immune subtyping system can be a guidance in the development of immunotherapy and personalized treatment of PCa patients.

## Data Availability

The data used to support the findings of this study are included within the article.

## Abbreviations

ADT: Androgen depravation therapy
CDF: Cumulative distribution function
CNV: Copy number variants
CTLA-4: Cytotoxic T-lymphocyte-associated protein 4
ESTIMATE: Estimation of STromal and Immune cells in MAlignant Tumours using Expression data
FDA: Food and Drug Administration
IS: Immune subtypes
LASSO: Least absolute shrinkage and selection operator
MCRPC: Metastatic castration-resistant prostate cancer
OS: Overall survival
PAM: Partitioning around medoids
PCa: Prostate cancer
PD-1: Programmed cell death protein 1
PD-L1: Programmed cell death ligand 1
PSA: Prostate-specific antigen
SsGSEA: Single-sample gene set enrichment analysis
TCGA: The Cancer Genome Atlas
TGF: Transforming growth factor
TIDE: Tumor immune dysfunction and exclusion
TIL: Tumor-infiltrating lymphocyte
TMB: Tumor mutation burden
TME: Tumor microenvironment
TOM: Topological overlap matrix
TPM: Transcripts per million
WGCNA: Weighted correlation network analysis.

## Supplementary Materials

Supplementary Materials
*Supplementary Figure S1.* Work flow chart. *Supplementary Figure S2*. Expression and interaction analysis of 11 genes. (A) Differential expression of 11 genes in cancer and adjacent tissues. (B) Correlation between 11 genes and immune infiltrating cells. (C) Correlation analysis between the expression of 11 genes and immune checkpoint genes. (D) Interaction between 11 genes. *Supplementary Table S1*. Immune related genes list. *Supplementary Table S2*. Clinical information of TCGA-PRAD dataset. *Supplementary Table S3*. Clinical information of MKSCC-PRAD dataset. *Supplementary Table S4*. Information of co-expression module corresponding to each gene.
