# Supplementary material for: Development of a Novel Immune Subtyping System Expanded with Immune Landscape and an 11-Gene Signature for Predicting Prostate Cancer Survival
Source: J Oncol. 2022 Feb 16;2022:1183173. doi: 10.1155/2022/1183173 (PMC8866019; doi:10.1155/2022/1183173)
Supplement: Supplementary Materials — Supplementary Figure S1. Work flow chart. Supplementary Figure S2. Expression and interaction analysis of 11 genes. (A) Differential expression of 11 genes in cancer and adjacent tissues. (B) Correlation between 11 genes and immune infiltrating cells. (C) Correlation analysis between the expression of 11 genes and immune checkpoint genes. (D) Interaction between 11 genes. Supplementary Table S1. Immune related genes list. Supplementary Table S2. Clinical information of TCGA-PRAD dataset. Supplementary Table S3. Clinical information of MKSCC-PRAD dataset. Supplementary Table S4. Information of co-expression module corresponding to each gene. [file 1183173.f1.zip › 1183173.f1/Supplementary Table S2 (1).pdf]

Supplementary Table S2. Clinical information of TCGA-PRAD dataset

Samples	OS.time	OS	DSS.time	DSS	DFI.time	DFI	PFI.time	PFI	Age	Gender	Stage	T_Stage	N_Stage	M_Stage	Cluster
TCGA-2A-A8VL	621	0	621	0	NA	NA	621	0	51	MALE	NA	T2b	N0	M0	IS4
TCGA-2A-A8VO	1701	0	1701	0	NA	NA	1701	0	57	MALE	NA	T1c		M0	IS4
TCGA-2A-A8VT	1373	0	1373	0	NA	NA	1373	0	47	MALE	NA	T2c	N1	M0	IS2
TCGA-2A-A8VV	671	0	671	0	NA	NA	671	0	52	MALE	NA	T2b	N0	M0	IS4
TCGA-2A-A8VX	1378	0	1378	0	NA	NA	1378	0	70	MALE	NA	T2c	N0	M0	IS2
TCGA-2A-A8W1	112	0	112	0	NA	NA	112	0	54	MALE	NA	T1c	N0	M0	IS2
TCGA-2A-A8W3	863	0	863	0	NA	NA	198	1	69	MALE	NA	T2b	N0	M0	IS2
TCGA-2A-AAYF	1364	0	1364	0	1364	0	1364	0	57	MALE	NA	T1c		M0	IS4
TCGA-2A-AAYO	1272	0	1272	0	1272	0	1272	0	57	MALE	NA	T1c		M0	IS1
TCGA-2A-AAYU	615	0	615	0	NA	NA	615	0	56	MALE	NA	T1c	N0	M0	IS4
TCGA-4L-AA1F	350	0	350	0	350	0	350	0	64	MALE	NA	T2c	N0	M1a	IS1
TCGA-CH-5737	91	0	91	0	NA	NA	91	0	73	MALE	NA	T2c	N0	M0	IS4
TCGA-CH-5738	212	0	212	0	212	0	212	0	72	MALE	NA			M0	IS3
TCGA-CH-5739	671	0	671	0	671	0	671	0	65	MALE	NA	T3a	N0	M0	IS3
TCGA-CH-5740	31	0	31	0	NA	NA	31	0	57	MALE	NA	T2c	N0	M0	IS2
TCGA-CH-5741	395	0	395	0	395	0	395	0	56	MALE	NA	T3b	N1	M0	IS2
TCGA-CH-5743	425	0	425	0	425	0	425	1	66	MALE	NA	T2c	N0	M0	IS1
TCGA-CH-5744	60	0	60	0	NA	NA	60	0	64	MALE	NA	T2c	N0	M0	IS2
TCGA-CH-5745	91	0	91	0	NA	NA	91	0	68	MALE	NA	T3a	N0	M0	IS3
TCGA-CH-5746	731	0	731	0	731	0	731	0	57	MALE	NA	T2c	N0	M0	IS4
TCGA-CH-5748	31	0	31	0	NA	NA	31	0	64	MALE	NA	T3a	N0		IS4
TCGA-CH-5750	396	0	396	0	396	0	396	0	72	MALE	NA	T2c	N0	M0	IS1
TCGA-CH-5751	1065	0	1065	0	365	1	365	1	68	MALE	NA	T4	N1	M0	IS1

TCGA-CH-5752	943	0	943	0	943	0	943	0	66	MALE	NA	T3a	N0	M0	IS3
TCGA-CH-5753	31	0	31	0	NA	NA	31	0	70	MALE	NA	T3b	N1	M0	IS1
TCGA-CH-5754	62	0	62	0	NA	NA	62	0	65	MALE	NA	T3b	N1	M0	IS1
TCGA-CH-5761	28	0	28	0	NA	NA	28	0	61	MALE	NA	T3b	N0	M0	IS2
TCGA-CH-5762	1339	0	1339	0	1339	0	1339	0	60	MALE	NA	T3b	N0	M0	IS3
TCGA-CH-5763	365	0	365	0	365	0	365	0	66	MALE	NA	T3a	N0	M0	IS3
TCGA-CH-5764	31	0	31	0	NA	NA	31	0	66	MALE	NA	T3b	N0	M0	IS3
TCGA-CH-5765	700	0	700	0	700	0	700	0	55	MALE	NA	T3a	N0	M0	IS4
TCGA-CH-5766	31	0	31	0	NA	NA	31	0	55	MALE	NA	T3a	N1	M0	IS1
TCGA-CH-5767	458	0	458	0	458	0	458	0	66	MALE	NA	T2c	N0	M0	IS4
TCGA-CH-5768	731	0	731	0	731	0	731	0	72	MALE	NA	T3a	N0	M0	IS4
TCGA-CH-5769	62	0	62	0	NA	NA	62	0	48	MALE	NA	T3b	N0	M0	IS3
TCGA-CH-5771	396	0	396	0	396	0	396	0	63	MALE	NA	T2c	N0	M0	IS3
TCGA-CH-5772	486	0	486	0	486	0	486	0	63	MALE	NA	T3a	N0	M0	IS3
TCGA-CH-5788	821	0	821	0	NA	NA	821	0	69	MALE	NA	T3b	N1	M0	IS2
TCGA-CH-5789	304	0	304	0	304	0	304	0	61	MALE	NA	T3a	N0	M0	IS3
TCGA-CH-5790	974	0	974	0	974	0	974	0	64	MALE	NA	T2c	N0	M0	IS4
TCGA-CH-5791	1004	0	1004	0	1004	0	396	1	72	MALE	NA	T3a	N0	M0	IS3
TCGA-CH-5792	91	0	91	0	NA	NA	91	0	57	MALE	NA	T3a	N0	M0	IS3
TCGA-CH-5794	882	0	882	0	882	0	882	0	65	MALE	NA	T2b	N0	M0	IS4
TCGA-EJ-5494	1477	0	1477	0	1477	0	1477	0	50	MALE	NA	T2a	N0	M0	IS3
TCGA-EJ-5495	1760	0	1760	0	1760	0	1760	0	68	MALE	NA	T1c	N1	M0	IS3
TCGA-EJ-5496	595	0	595	0	595	0	595	0	59	MALE	NA	T1c	N0	M0	IS4
TCGA-EJ-5497	405	0	405	0	405	0	405	0	47	MALE	NA	T2c	N0	M0	IS3
TCGA-EJ-5498	1720	0	1720	0	1720	0	1720	0	56	MALE	NA	T2c	N0		IS3

TCGA-EJ-5499	1415	0	1415	0	1415	0	1415	0	61	MALE	NA	T2b	N0	M0	IS3
TCGA-EJ-5501	1365	0	1365	0	1365	0	1365	0	55	MALE	NA	T2b	N0	M0	IS4
TCGA-EJ-5502	1171	0	1171	0	1171	0	1171	0	50	MALE	NA	T2c	N0	M0	IS3
TCGA-EJ-5503	1391	0	1391	0	1391	0	1391	0	50	MALE	NA	T2b	N0	M0	IS3
TCGA-EJ-5504	1427	0	1427	0	NA	NA	1427	0	65	MALE	NA	T2c	N1	M0	IS3
TCGA-EJ-5505	922	0	922	0	922	0	922	0	57	MALE	NA	T1c	N0	M0	IS4
TCGA-EJ-5506	1381	0	1381	0	1381	0	1381	0	67	MALE	NA	T3b	N1	M0	IS4
TCGA-EJ-5507	1457	0	1457	0	1457	0	1457	0	54	MALE	NA	T2c	N1	M0	IS3
TCGA-EJ-5508	1965	0	1965	0	1965	0	1965	0	65	MALE	NA	T1c	N0	M0	IS3
TCGA-EJ-5509	1464	0	1464	0	1464	0	1464	0	63	MALE	NA	T2a	N0	M0	IS3
TCGA-EJ-5510	1887	0	1887	0	1887	0	1887	0	48	MALE	NA	T1c	N0	M0	IS3
TCGA-EJ-5511	1476	0	1476	0	1476	0	1476	0	55	MALE	NA	T2b	N0	M0	IS4
TCGA-EJ-5512	1733	0	1733	0	1733	0	1733	0	46	MALE	NA	T1c	N0	M0	IS4
TCGA-EJ-5514	1829	0	1829	0	1829	0	1829	0	66	MALE	NA	T1c	N0	M0	IS3
TCGA-EJ-5515	1832	0	1832	0	1832	0	1832	0	60	MALE	NA	T1c	N0	M0	IS4
TCGA-EJ-5516	1889	0	1889	0	1889	0	1889	0	49	MALE	NA	T2b	N0	M0	IS3
TCGA-EJ-5517	1889	0	1889	0	1889	0	1889	0	55	MALE	NA	T1c	N0	M0	IS3
TCGA-EJ-5518	2118	0	2118	0	NA	NA	2104	1	66	MALE	NA	T3b	N0	M0	IS3
TCGA-EJ-5519	1962	0	1962	0	1962	0	1962	0	64	MALE	NA	T2c	N1	M0	IS3
TCGA-EJ-5521	2279	0	2279	0	2279	0	2279	0	63	MALE	NA	T1c	N0	M0	IS3
TCGA-EJ-5522	2079	0	2079	0	2079	0	2079	0	51	MALE	NA	T2a	N0	M0	IS3
TCGA-EJ-5524	1882	0	1882	0	NA	NA	265	1	57	MALE	NA	T2b	N0	M0	IS3
TCGA-EJ-5525	1115	0	1115	0	NA	NA	546	1	67	MALE	NA	T3a	N0	M0	IS3
TCGA-EJ-5526	1909	0	1909	0	NA	NA	537	1	56	MALE	NA	T1c	N1	M0	IS3
TCGA-EJ-5527	1778	0	1778	0	1778	0	1778	0	69	MALE	NA	T2a	N0	M0	IS3

TCGA-EJ-5530	1832	0	1832	0	1832	0	1832	0	61	MALE	NA	T2c	N0	M0	IS3
TCGA-EJ-5531	1273	0	1273	0	1273	0	1273	0	62	MALE	NA	T2b	N0	M0	IS3
TCGA-EJ-5532	1828	0	1828	0	1828	0	1828	0	57	MALE	NA	T1c	N0	M0	IS3
TCGA-EJ-5542	1513	0	1513	0	1513	0	1513	0	60	MALE	NA	T2b	N0	M0	IS3
TCGA-EJ-7115	2687	0	2687	0	2687	0	2687	0	65	MALE	NA	T1c	N0	M0	IS3
TCGA-EJ-7123	2572	0	2572	0	2572	0	2572	0	59	MALE	NA	T1c	N0	M0	IS4
TCGA-EJ-7125	2850	0	2850	0	2850	0	2850	0	44	MALE	NA	T1c	N0	M0	IS3
TCGA-EJ-7218	2542	0	2542	0	2542	0	2542	0	71	MALE	NA	T2a	N0	M0	IS4
TCGA-EJ-7312	1099	0	1099	0	1099	0	1099	0	58	MALE	NA	T3b	N0	M0	IS2
TCGA-EJ-7314	1150	0	1150	0	1150	0	1150	0	62	MALE	NA	T2c	N0	M0	IS4
TCGA-EJ-7315	1008	0	1008	0	1008	0	1008	0	68	MALE	NA	T2a	N0	M0	IS1
TCGA-EJ-7317	771	0	771	0	771	0	771	0	71	MALE	NA	T1c	N0	M0	IS4
TCGA-EJ-7318	1029	0	1029	0	NA	NA	380	1	51	MALE	NA	T3a		M0	IS1
TCGA-EJ-7321	824	0	824	0	824	0	824	0	57	MALE	NA	T1c	N0	M0	IS1
TCGA-EJ-7325	1070	0	1070	0	1070	0	1070	0	63	MALE	NA	T3b	N0	M0	IS2
TCGA-EJ-7327	923	0	923	0	923	0	923	0	61	MALE	NA	T2a	N0	M0	IS3
TCGA-EJ-7328	842	0	842	0	842	0	842	0	70	MALE	NA	T1c	N0	M0	IS1
TCGA-EJ-7330	191	0	191	0	NA	NA	191	0	68	MALE	NA	T1c	N0	M0	IS3
TCGA-EJ-7331	742	0	742	0	742	0	742	0	64	MALE	NA	T1c	N0	M0	IS3
TCGA-EJ-7781	1073	0	1073	0	1073	0	1073	0	65	MALE	NA	T1c	N0	M0	IS3
TCGA-EJ-7782	1167	0	1167	0	1167	0	1167	0	71	MALE	NA	T2c	N0	M0	IS3
TCGA-EJ-7783	787	0	787	0	787	0	787	0	70	MALE	NA	T2c	N0	M0	IS3
TCGA-EJ-7784	781	0	781	0	781	0	781	0	63	MALE	NA	T2c	N0	M0	IS4
TCGA-EJ-7785	1177	0	1177	0	1177	0	1177	0	54	MALE	NA	T2b	N0	M0	IS3
TCGA-EJ-7786	1096	0	1096	0	1096	0	1096	0	62	MALE	NA	T1c	N0	M0	IS4

TCGA-EJ-7788	859	0	859	0	859	0	859	0	53	MALE	NA	T3b	N0	M0	IS3
TCGA-EJ-7789	1105	0	1105	0	1105	0	1105	0	66	MALE	NA	T1c	N0	M0	IS2
TCGA-EJ-7791	1118	0	1118	0	1118	0	1118	0	67	MALE	NA	T1c	N0	M0	IS3
TCGA-EJ-7792	1396	0	1396	0	1396	0	1396	0	53	MALE	NA	T1c	N0	M0	IS3
TCGA-EJ-7793	114	0	114	0	114	0	114	0	49	MALE	NA	T2c		M0	IS4
TCGA-EJ-7794	1306	0	1306	0	1306	0	1306	0	67	MALE	NA	T2c	N0	M0	IS3
TCGA-EJ-7797	983	0	983	0	983	0	983	0	53	MALE	NA	T1c	N0	M0	IS4
TCGA-EJ-8468	2347	0	2347	0	2347	0	2347	0	63	MALE	NA	T3a	N0		IS3
TCGA-EJ-8469	2324	0	2324	0	NA	NA	1925	1	46	MALE	NA	T3a	N0	M0	IS2
TCGA-EJ-8470	1159	0	1159	0	1159	0	1159	0	58	MALE	NA	T2c	N0	M0	IS1
TCGA-EJ-8472	756	0	756	0	NA	NA	196	1	63	MALE	NA	T3a	N0	M0	IS2
TCGA-EJ-8474	766	0	766	0	766	0	766	0	69	MALE	NA	T3a	N0	M0	IS3
TCGA-EJ-A46B	657	0	657	0	657	0	657	0	66	MALE	NA	T1c	N0	M0	IS4
TCGA-EJ-A46D	626	0	626	0	626	0	626	0	53	MALE	NA	T2c	N0	M0	IS4
TCGA-EJ-A46E	524	0	524	0	524	0	524	0	57	MALE	NA	T1c	N0	M0	IS4
TCGA-EJ-A46F	783	0	783	0	NA	NA	215	1	57	MALE	NA	T2c	N1	M0	IS2
TCGA-EJ-A46G	668	0	668	0	668	0	668	0	71	MALE	NA	T3a	N0	M0	IS1
TCGA-EJ-A46H	724	0	724	0	724	0	724	0	60	MALE	NA	T1c	N0	M0	IS3
TCGA-EJ-A46I	668	0	668	0	668	0	668	0	57	MALE	NA	T1c	N0	M0	IS4
TCGA-EJ-A65B	710	0	710	0	710	0	710	0	55	MALE	NA	T3b	N0	M0	IS2
TCGA-EJ-A65D	393	0	393	0	393	0	393	0	66	MALE	NA	T3a	N0	M0	IS2
TCGA-EJ-A65E	417	0	417	0	417	0	417	0	67	MALE	NA	T3b	N0	M0	IS2
TCGA-EJ-A65F	560	0	560	0	NA	NA	75	1	59	MALE	NA	T3b	N0	M0	IS3
TCGA-EJ-A65G	666	0	666	0	666	0	666	0	57	MALE	NA	T2c	N0	M0	IS4
TCGA-EJ-A65J	451	0	451	0	451	0	451	0	62	MALE	NA	T3a	N0	M0	IS2

TCGA-EJ-A65M	230	0	230	0	230	0	230	0	65	MALE	NA	T2c	N0	M0	IS1
TCGA-EJ-A6RA	353	0	353	0	NA	NA	353	1	70	MALE	NA	T3b	N1	M0	IS4
TCGA-EJ-A6RC	873	0	873	0	873	0	873	0	64	MALE	NA	T1c	N0	M0	IS3
TCGA-EJ-A7NF	250	0	250	0	250	0	250	0	56	MALE	NA	T2c	N0	M0	IS2
TCGA-EJ-A7NG	432	0	432	0	432	0	432	0	64	MALE	NA	T3a	N0	M0	IS4
TCGA-EJ-A7NH	474	0	474	0	474	0	474	0	56	MALE	NA	T2c	N0	M0	IS4
TCGA-EJ-A7NJ	197	0	197	0	197	0	197	0	59	MALE	NA	T2c	N0	M0	IS4
TCGA-EJ-A7NK	476	0	476	0	476	0	476	0	58	MALE	NA	T3a	N0	M0	IS4
TCGA-EJ-A7NM	128	0	128	0	128	0	128	0	64	MALE	NA	T3b	N1	M0	IS1
TCGA-EJ-A7NN	197	0	197	0	197	1	197	1	61	MALE	NA	T3b	N1	M0	IS1
TCGA-EJ-A8FN	329	0	329	0	329	0	329	0	58	MALE	NA	T2c	N0	M0	IS3
TCGA-EJ-A8FO	282	0	282	0	282	0	282	0	51	MALE	NA	T3a	N0	M0	IS4
TCGA-EJ-A8FP	299	0	299	0	117	1	117	1	59	MALE	NA	T2c	N0	M0	IS3
TCGA-EJ-A8FS	266	0	266	0	216	1	216	1	66	MALE	NA	T3a	N0	M0	IS2
TCGA-EJ-A8FU	138	0	138	0	138	0	138	0	64	MALE	NA	T3a	N0	M0	IS3
TCGA-EJ-AB20	131	0	131	0	131	0	131	0	61	MALE	NA	T2c	N0	M0	IS3
TCGA-EJ-AB27	145	0	145	0	145	0	145	0	51	MALE	NA	T1c	N0	M0	IS4
TCGA-FC-7708	864	0	864	0	864	0	864	0	52	MALE	NA	T1c	N0	M0	IS3
TCGA-FC-7961	469	0	469	0	469	0	469	0	62	MALE	NA	T1c	N0	M0	IS3
TCGA-FC-A4JI	877	0	877	0	877	0	877	0	70	MALE	NA	T4		M0	IS2
TCGA-FC-A5OB	680	0	680	0	NA	NA	680	0	53	MALE	NA	T3a	N0	M0	IS4
TCGA-FC-A66V	524	0	524	0	524	0	524	0	74	MALE	NA	T3b	N0	M0	IS2
TCGA-FC-A6HD	789	0	789	0	789	0	789	0	77	MALE	NA	T3a	N0	M0	IS2
TCGA-FC-A8O0	616	0	616	0	616	0	616	0	68	MALE	NA	T2c	N0	M0	IS4
TCGA-G9-6329	1266	0	1266	0	1266	0	1266	0	67	MALE	NA	T1c		M0	IS3

TCGA-G9-6332	2657	0	2657	0	1180	1	1180	1	55	MALE	NA	T2b	N0	M0	IS2
TCGA-G9-6333	2465	0	2465	0	2465	0	2465	0	66	MALE	NA	T1c	N0	M0	IS4
TCGA-G9-6336	2068	0	2068	0	2068	0	2068	0	57	MALE	NA	T2c	N0	M0	IS4
TCGA-G9-6338	2028	0	2028	0	2028	0	2028	0	61	MALE	NA	T1c	N0	M0	IS4
TCGA-G9-6339	2551	0	2551	0	1634	1	1634	1	58	MALE	NA	T3b	N0	M0	IS4
TCGA-G9-6342	1696	0	1696	0	1696	0	1696	0	61	MALE	NA	T2c		M0	IS4
TCGA-G9-6343	2450	0	2450	0	2450	0	2450	0	62	MALE	NA	T2a		M0	IS4
TCGA-G9-6347	2089	0	2089	0	2089	0	2089	0	59	MALE	NA	T1c		M0	IS1
TCGA-G9-6348	1515	0	1515	0	1515	0	1515	0	68	MALE	NA	T3a	N0	M0	IS3
TCGA-G9-6351	2048	0	2048	0	2048	0	2048	0	51	MALE	NA	T1c		M0	IS4
TCGA-G9-6353	1542	0	1542	0	1542	0	1542	0	58	MALE	NA	T1c		M0	IS4
TCGA-G9-6354	2066	0	2066	0	2066	0	2066	0	55	MALE	NA	T1c		M0	IS4
TCGA-G9-6356	1434	0	1434	0	1434	0	1434	0	60	MALE	NA	T1c	N0	M0	IS3
TCGA-G9-6361	1415	0	1415	0	1415	0	1415	0	61	MALE	NA	T2c	N0	M0	IS4
TCGA-G9-6362	1443	0	1443	0	NA	NA	1443	0	57	MALE	NA	T1c	N0	M0	IS2
TCGA-G9-6363	1378	0	1378	0	1378	0	1378	0	64	MALE	NA	T2b	N0	M0	IS4
TCGA-G9-6364	1198	0	1198	0	1198	0	1198	0	72	MALE	NA	T3a	N0	M0	IS3
TCGA-G9-6365	1363	0	1363	0	1363	0	1363	0	71	MALE	NA	T2a	N0		IS3
TCGA-G9-6366	1947	0	1947	0	1947	0	1947	0	61	MALE	NA	T1c			IS4
TCGA-G9-6367	1222	0	1222	0	NA	NA	1222	0	60	MALE	NA	T2b	N0	M0	IS4
TCGA-G9-6369	1215	0	1215	0	1215	0	1215	0	55	MALE	NA	T1c	N0	M0	IS4
TCGA-G9-6370	1156	0	1156	0	1156	0	1156	0	52	MALE	NA	T1c	N0	M0	IS3
TCGA-G9-6371	1226	0	1226	0	1226	0	1226	0	58	MALE	NA	T1c		M0	IS4
TCGA-G9-6373	811	0	811	0	811	0	811	0	68	MALE	NA	T2b		M0	IS4
TCGA-G9-6377	958	0	958	0	958	0	958	0	61	MALE	NA	T2b	N0	M0	IS4

TCGA-G9-6378	1155	0	1155	0	1155	0	1155	0	54	MALE	NA	T1c		M0	IS3
TCGA-G9-6379	1778	0	1778	0	1778	0	1778	0	68	MALE	NA	T1b	N0	M0	IS1
TCGA-G9-6384	765	0	765	0	765	0	765	0	53	MALE	NA	T1c	N0	M0	IS3
TCGA-G9-6385	830	0	830	0	830	0	830	0	66	MALE	NA	T1c		M0	IS4
TCGA-G9-6494	1771	0	1771	0	NA	NA	1771	0	66	MALE	NA	T1c	N0	M0	IS3
TCGA-G9-6496	1726	0	1726	0	1726	0	1726	0	61	MALE	NA	T1c	N0	M0	IS2
TCGA-G9-6498	1952	0	1952	0	1342	1	1342	1	53	MALE	NA	T1c		M0	IS1
TCGA-G9-6499	1543	0	1543	0	1543	0	1543	0	66	MALE	NA	T3a	N0	M0	IS4
TCGA-G9-7509	1610	0	1610	0	1610	0	1610	0	48	MALE	NA	T1c		M0	IS4
TCGA-G9-7510	1185	0	1185	0	1185	0	1185	0	66	MALE	NA	T1c	N0	M0	IS3
TCGA-G9-7519	849	0	849	0	849	0	849	0	58	MALE	NA	T1c		M0	IS4
TCGA-G9-7521	942	0	942	0	NA	NA	942	0	52	MALE	NA	T3a	N1	M0	IS1
TCGA-G9-7522	1078	0	1078	0	1078	0	1078	0	49	MALE	NA	T1c	N0	M0	IS3
TCGA-G9-7523	857	0	857	0	857	0	857	0	44	MALE	NA	T1c	N0	M0	IS3
TCGA-G9-7525	994	0	994	0	994	0	994	0	64	MALE	NA	T1c	N0	M0	IS4
TCGA-G9-A9S0	791	1	791	1	NA	NA	423	1	53	MALE	NA	T2a	N1	M0	IS1
TCGA-G9-A9S4	906	0	906	0	906	0	906	0	62	MALE	NA	T2a	N1	M0	IS2
TCGA-G9-A9S7	728	0	728	0	NA	NA	728	0	55	MALE	NA	T2a	N0	M0	IS2
TCGA-H9-7775	185	0	185	0	185	0	185	0	68	MALE	NA	T2c			IS4
TCGA-H9-A6BX	941	0	941	0	941	0	941	0	60	MALE	NA	T2c			IS3
TCGA-H9-A6BY	112	0	112	0	NA	NA	112	0	63	MALE	NA	T1c	N0		IS2
TCGA-HC-7075	601	0	601	0	601	0	601	0	63	MALE	NA	T1c		M0	IS4
TCGA-HC-7077	2115	0	2115	0	2115	0	2115	0	64	MALE	NA	T1c		M0	IS2
TCGA-HC-7078	1985	0	1985	0	1985	0	1985	0	69	MALE	NA	T1c	N0	M0	IS1
TCGA-HC-7079	1184	0	1184	0	NA	NA	380	1	51	MALE	NA	T3a	N0	M0	IS1

TCGA-HC-7080	1106	0	1106	0	1106	0	894	1	66	MALE	NA	T2a	N0	M0	IS2
TCGA-HC-7081	1136	0	1136	0	NA	NA	1136	0	62	MALE	NA	T3b			IS3
TCGA-HC-7209	440	0	440	0	440	0	440	0	60	MALE	NA	T2b	N0		IS3
TCGA-HC-7210	868	0	868	0	868	0	868	0	74	MALE	NA	T2a	N0		IS3
TCGA-HC-7211	1221	0	1221	0	1221	0	1221	0	62	MALE	NA	T2a	N0	M0	IS3
TCGA-HC-7212	870	0	870	0	870	0	870	0	56	MALE	NA	T2a	N0	M0	IS3
TCGA-HC-7213	1219	0	1219	0	NA	NA	170	1	53	MALE	NA	T3b	N0	M0	IS2
TCGA-HC-7230	1001	0	1001	0	1001	0	1001	0	50	MALE	NA	T2c	N0		IS4
TCGA-HC-7231	939	0	939	0	939	0	939	0	66	MALE	NA	T1c		M0	IS1
TCGA-HC-7232	1161	0	1161	0	766	1	766	1	66	MALE	NA	T3b			IS3
TCGA-HC-7233	1355	0	1355	0	1355	0	1355	0	73	MALE	NA	T2a	N0		IS3
TCGA-HC-7736	1103	0	1103	0	1103	0	1103	0	59	MALE	NA	T2b	N0	M0	IS3
TCGA-HC-7737	1090	0	1090	0	1090	0	1090	0	55	MALE	NA	T1c	N1	M0	IS3
TCGA-HC-7738	964	0	964	0	NA	NA	420	1	58	MALE	NA	T2c		M0	IS4
TCGA-HC-7740	717	0	717	0	717	0	717	0	59	MALE	NA	T2a	N0	M0	IS3
TCGA-HC-7742	938	0	938	0	NA	NA	938	0	58	MALE	NA	T3a	N0	M0	IS3
TCGA-HC-7744	531	0	531	0	531	0	531	0	46	MALE	NA	T3b	N0	M0	IS4
TCGA-HC-7745	721	0	721	0	721	0	721	0	65	MALE	NA	T3b	N0		IS3
TCGA-HC-7747	748	0	748	0	748	0	748	0	52	MALE	NA	T2b	N0	M0	IS3
TCGA-HC-7748	792	0	792	0	792	0	792	0	60	MALE	NA	T2a	N0		IS3
TCGA-HC-7749	724	0	724	0	724	0	724	0	70	MALE	NA	T3a	N0	M0	IS3
TCGA-HC-7750	522	0	522	0	522	0	522	0	50	MALE	NA	T2b		M0	IS4
TCGA-HC-7752	782	0	782	0	782	0	782	0	61	MALE	NA	T2c	N0	M0	IS1
TCGA-HC-7817	928	0	928	0	928	0	928	0	52	MALE	NA	T3b		M0	IS3
TCGA-HC-7818	938	0	938	0	938	0	938	0	66	MALE	NA	T2b	N0	M0	IS3

TCGA-HC-7819	543	0	543	0	543	0	543	0	53	MALE	NA	T2c	N0	M0	IS4
TCGA-HC-7820	747	0	747	0	747	0	747	0	72	MALE	NA	T2c			IS4
TCGA-HC-7821	956	0	956	0	956	0	956	0	54	MALE	NA	T3b	N1	M0	IS1
TCGA-HC-8213	589	0	589	0	589	0	589	0	48	MALE	NA	T2c			IS4
TCGA-HC-8216	682	0	682	0	682	0	682	0	58	MALE	NA	T2a	N0	M0	IS1
TCGA-HC-8256	860	0	860	0	860	0	860	0	53	MALE	NA	T2c		M0	IS4
TCGA-HC-8257	726	0	726	0	726	0	726	0	70	MALE	NA	T3b	N1		IS1
TCGA-HC-8258	847	0	847	0	847	0	847	0	56	MALE	NA	T2c	N0		IS4
TCGA-HC-8259	924	0	924	0	924	0	924	0	50	MALE	NA	T1c		M0	IS4
TCGA-HC-8260	686	0	686	0	686	0	686	0	43	MALE	NA	T2c	N0	M0	IS4
TCGA-HC-8261	546	0	546	0	546	0	546	0	48	MALE	NA	T2c			IS1
TCGA-HC-8262	679	0	679	0	679	0	679	0	57	MALE	NA	T2c	N0		IS4
TCGA-HC-8264	48	0	48	0	NA	NA	48	0	60	MALE	NA	T3b	N1		IS3
TCGA-HC-8265	483	0	483	0	483	0	483	0	66	MALE	NA	T3a	N0		IS2
TCGA-HC-8266	473	0	473	0	NA	NA	473	0	55	MALE	NA	T3b	N0	M0	IS1
TCGA-HC-A48F	680	0	680	0	680	0	680	0	47	MALE	NA	T1c	N1	M0	IS2
TCGA-HC-A4ZV	23	0	23	0	NA	NA	23	0	56	MALE	NA	T2c	N0		IS1
TCGA-HC-A631	54	0	54	0	NA	NA	54	0	67	MALE	NA	T2	N0	M0	IS2
TCGA-HC-A632	61	0	61	0	NA	NA	61	0	68	MALE	NA	T1c	N0	M0	IS1
TCGA-HC-A6AL	67	0	67	0	NA	NA	67	0	64	MALE	NA	T1c	N0	M0	IS1
TCGA-HC-A6AN	49	0	49	0	NA	NA	49	0	67	MALE	NA	T1c	N0	M0	IS2
TCGA-HC-A6AO	530	0	530	0	530	0	530	0	52	MALE	NA	T1c		M0	IS1
TCGA-HC-A6AP	71	0	71	0	NA	NA	71	0	61	MALE	NA	T1c		M0	IS1
TCGA-HC-A6AQ	106	0	106	0	NA	NA	106	0	60	MALE	NA	T1c		M0	IS2
TCGA-HC-A6AS	44	0	44	0	NA	NA	44	0	70	MALE	NA	T1c	N0	M0	IS1

TCGA-HC-A6HX	38	0	38	0	NA	NA	38	0	55	MALE	NA	T1c	N0	M0	IS2
TCGA-HC-A6HY	134	0	134	0	NA	NA	134	0	51	MALE	NA	T1c		M0	IS2
TCGA-HC-A76W	213	0	213	0	NA	NA	213	0	73	MALE	NA	T2	N0	M0	IS3
TCGA-HC-A76X	150	0	150	0	NA	NA	150	0	62	MALE	NA	T1c	N0	M0	IS4
TCGA-HC-A8CY	290	0	290	0	NA	NA	290	0	63	MALE	NA	T1c	N1	M0	IS1
TCGA-HC-A8D0	775	0	775	0	775	0	775	0	61	MALE	NA	T1c	N0	M0	IS1
TCGA-HC-A8D1	573	0	573	0	573	0	573	0	68	MALE	NA	T2	N0	M0	IS3
TCGA-HC-A9TE	587	0	587	0	NA	NA	216	1	64	MALE	NA	T2c	N0	M0	IS1
TCGA-HC-A9TH	844	0	844	0	NA	NA	351	1	58	MALE	NA	T1b	N0	M0	IS1
TCGA-HI-7168	3323	0	3323	0	NA	NA	2505	1	62	MALE	NA	T1c	N0	M0	IS3
TCGA-HI-7169	2684	0	2684	0	2684	0	2684	0	55	MALE	NA	T2b	N0	M0	IS4
TCGA-HI-7170	2522	0	2522	0	2522	0	2522	0	58	MALE	NA	T1c	N0	M0	IS3
TCGA-HI-7171	1329	1	1329	1	NA	NA	1329	1	56	MALE	NA	T3a	N0	M0	IS2
TCGA-J4-8198	614	0	614	0	614	0	614	0	49	MALE	NA	T1c		M0	IS4
TCGA-J4-8200	1218	0	1218	0	1218	0	1218	0	47	MALE	NA	T2	N0	M0	IS4
TCGA-J4-A67K	1004	0	1004	0	1004	0	1004	0	68	MALE	NA	T2b		M0	IS1
TCGA-J4-A67L	770	0	770	0	770	0	770	0	54	MALE	NA	T2a	N0	M0	IS1
TCGA-J4-A67M	941	0	941	0	941	0	941	0	55	MALE	NA	T2a	N0	M0	IS1
TCGA-J4-A67N	937	0	937	0	NA	NA	442	1	61	MALE	NA	T2a	N0	M0	IS2
TCGA-J4-A67O	839	0	839	0	839	0	839	0	59	MALE	NA	T2a	N0	M0	IS4
TCGA-J4-A67Q	975	0	975	0	975	0	975	0	77	MALE	NA	T2b		M0	IS1
TCGA-J4-A67R	910	0	910	0	910	0	910	0	65	MALE	NA	T1c	N0	M0	IS1
TCGA-J4-A67S	833	0	833	0	708	1	708	1	63	MALE	NA	T2b	N0	M0	IS2
TCGA-J4-A67T	183	0	183	0	183	0	183	0	63	MALE	NA	T1c		M0	IS4
TCGA-J4-A6G1	769	0	769	0	769	0	769	0	68	MALE	NA	T2b	N0	M0	IS1

TCGA-J4-A6G3	849	0	849	0	NA	NA	618	1	57	MALE	NA	T2	N0	M0	IS1
TCGA-J4-A6M7	512	0	512	0	512	0	512	0	53	MALE	NA	T2		M0	IS4
TCGA-J4-A83I	685	0	685	0	685	0	685	0	63	MALE	NA	T1c	N0	M0	IS2
TCGA-J4-A83J	696	0	696	0	696	0	696	0	68	MALE	NA	T1c	N0	M0	IS1
TCGA-J4-A83K	328	0	328	0	328	0	328	0	52	MALE	NA	T1c		M0	IS4
TCGA-J4-A83L	720	0	720	0	720	0	720	0	61	MALE	NA	T1c	N0	M0	IS4
TCGA-J4-A83M	543	0	543	0	521	1	521	1	64	MALE	NA	T2c	N0	M0	IS2
TCGA-J4-A83N	992	0	992	0	992	0	433	1	57	MALE	NA	T1c		M0	IS4
TCGA-J4-AATV	553	0	553	0	NA	NA	553	0	71	MALE	NA	T2c	N0	M0	IS1
TCGA-J4-AATZ	412	0	412	0	79	1	79	1	66	MALE	NA	T3a	N0	M0	IS2
TCGA-J4-AAU2	825	0	825	0	825	0	825	0	59	MALE	NA	T2c	N0	M0	IS4
TCGA-J9-A52B	422	0	422	0	57	1	57	1	62	MALE	NA	T3b	N0		IS2
TCGA-J9-A52C	178	0	178	0	NA	NA	178	0	57	MALE	NA	T1c			IS1
TCGA-J9-A52D	212	0	212	0	NA	NA	212	0	71	MALE	NA	T2b		M0	IS2
TCGA-J9-A52E	323	0	323	0	NA	NA	323	0	65	MALE	NA	T2c	N0		IS1
TCGA-J9-A8CK	321	0	321	0	NA	NA	321	0	66	MALE	NA	T3a		M0	IS2
TCGA-J9-A8CL	405	0	405	0	NA	NA	132	1	66	MALE	NA	T3a		M0	IS1
TCGA-J9-A8CM	463	0	463	0	NA	NA	344	1	66	MALE	NA	T2c	N1	M0	IS3
TCGA-J9-A8CN	1237	0	1237	0	1237	0	1237	0	53	MALE	NA	T1c		M0	IS2
TCGA-J9-A8CP	386	0	386	0	386	0	386	0	66	MALE	NA	T2a		M0	IS2
TCGA-KC-A4BL	934	0	934	0	NA	NA	193	1	65	MALE	NA	T1c	N0	M0	IS1
TCGA-KC-A4BN	1815	0	1815	0	1815	0	1815	0	55	MALE	NA	T1c	N0	M0	IS4
TCGA-KC-A4BR	1349	0	1349	0	1022	1	1022	1	75	MALE	NA	T2	N1	M0	IS4
TCGA-KC-A4BV	1328	0	1328	0	1328	1	1328	1	66	MALE	NA	T2	N0	M0	IS3
TCGA-KC-A7F3	665	0	665	0	665	0	665	0	67	MALE	NA	T1c	N0	M0	IS4

TCGA-KC-A7F5	91	0	91	0	91	0	91	0	54	MALE	NA	T2c	N0	M0	IS2
TCGA-KC-A7F6	263	0	263	0	263	0	263	0	63	MALE	NA	T1c	N0	M0	IS2
TCGA-KC-A7FA	598	0	598	0	598	0	598	0	63	MALE	NA	T2c	N0	M0	IS2
TCGA-KC-A7FD	270	0	270	0	270	0	270	0	62	MALE	NA	T1c	N0	M0	IS2
TCGA-KC-A7FE	352	0	352	0	352	0	352	0	62	MALE	NA	T1c	N0	M0	IS3
TCGA-KK-A59V	3440	0	3440	0	3440	0	3440	0	64	MALE	NA	T3a	N0	M0	IS1
TCGA-KK-A59X	2535	0	2535	0	NA	NA	2233	1	55	MALE	NA	T3a	N1	M0	IS2
TCGA-KK-A59Y	1968	0	1968	0	1968	0	1968	0	56	MALE	NA	T3a	N1	M0	IS2
TCGA-KK-A59Z	2628	0	2628	0	NA	NA	2628	0	66	MALE	NA	T2b	N0	M0	IS3
TCGA-KK-A5A1	2469	1	2469	NA	NA	NA	203	1	72	MALE	NA	T3a	N0	M0	IS1
TCGA-KK-A6DY	4295	0	4295	0	NA	NA	4295	0	50	MALE	NA	T1c	N0	M0	IS1
TCGA-KK-A6E0	1483	0	1483	0	941	1	941	1	59	MALE	NA	T2b	N0	M0	IS2
TCGA-KK-A6E1	2198	0	2198	0	2198	0	2198	0	57	MALE	NA	T2c	N1	M0	IS2
TCGA-KK-A6E2	5024	0	5024	0	5024	0	5024	0	54	MALE	NA	T2a	N0	M0	IS2
TCGA-KK-A6E3	2056	0	2056	0	NA	NA	2056	0	56	MALE	NA	T1c	N0	M0	IS4
TCGA-KK-A6E4	3502	1	3502	0	3502	0	3502	0	69	MALE	NA	T1c	N0	M0	IS2
TCGA-KK-A6E5	2073	0	2073	0	2073	0	2073	0	63	MALE	NA	T1c	N0	M0	IS4
TCGA-KK-A6E6	3447	0	3447	0	NA	NA	3447	0	69	MALE	NA	T2b	N0	M0	IS3
TCGA-KK-A6E7	2760	0	2760	0	NA	NA	925	1	46	MALE	NA	T1c	N1	M0	IS2
TCGA-KK-A6E8	1770	0	1770	0	NA	NA	1770	0	68	MALE	NA	T1c	N0	M0	IS2
TCGA-KK-A7AP	196	0	196	0	NA	NA	196	0	55	MALE	NA	T2b	N1	M0	IS2
TCGA-KK-A7AQ	1610	0	1610	0	1217	1	1217	1	62	MALE	NA	T2a	N0	M0	IS2
TCGA-KK-A7AU	1775	0	1775	0	NA	NA	207	1	63	MALE	NA	T2b	N1	M0	IS2
TCGA-KK-A7AV	822	0	822	0	822	0	822	0	58	MALE	NA	T1c	N0	M0	IS1
TCGA-KK-A7AW	1054	0	1054	0	NA	NA	1054	0	57	MALE	NA	T2a	N1	M0	IS1

TCGA-KK-A7AY	1754	0	1754	0	1124	1	1124	1	60	MALE	NA	T2a	N0	M0	IS2
TCGA-KK-A7AZ	1519	0	1519	0	NA	NA	1519	0	56	MALE	NA	T2b	N0		IS2
TCGA-KK-A7B0	1329	0	1329	0	NA	NA	606	1	67	MALE	NA	T2b	N0	M0	IS1
TCGA-KK-A7B1	1063	0	1063	0	1063	0	1063	0	65	MALE	NA	T1c	N0	M0	IS2
TCGA-KK-A7B2	1099	0	1099	0	NA	NA	692	1	68	MALE	NA	T1c	N1	M0	IS1
TCGA-KK-A7B3	899	0	899	0	294	1	294	1	62	MALE	NA	T3a	N0	M0	IS1
TCGA-KK-A7B4	987	0	987	0	NA	NA	637	1	64	MALE	NA	T3b	N1	M0	IS1
TCGA-KK-A8I4	2259	0	2259	0	NA	NA	1162	1	64	MALE	NA	T2c	N1	M0	IS1
TCGA-KK-A8I5	3096	0	3096	0	3096	0	3096	0	55	MALE	NA	T2a	N0	M0	IS4
TCGA-KK-A8I6	668	0	668	0	668	0	668	0	58	MALE	NA	T2b	N0	M0	IS2
TCGA-KK-A8I7	2002	0	2002	0	NA	NA	1088	1	55	MALE	NA	T2c	N0	M0	IS3
TCGA-KK-A8I8	967	0	967	0	967	0	967	0	69	MALE	NA	T2a	N0	M0	IS2
TCGA-KK-A8I9	1001	0	1001	0	940	1	940	1	61	MALE	NA	T2b	N0	M0	IS1
TCGA-KK-A8IA	1931	0	1931	0	NA	NA	1931	0	69	MALE	NA	T3b	N1	M0	IS2
TCGA-KK-A8IB	83	0	83	0	NA	NA	83	0	65	MALE	NA	T2a	N0	M0	IS1
TCGA-KK-A8IC	2051	0	2051	0	NA	NA	1060	1	54	MALE	NA	T1c	N0	M0	IS3
TCGA-KK-A8ID	2107	0	2107	0	2107	0	2107	0	70	MALE	NA	T2b	N1	M0	IS2
TCGA-KK-A8IF	804	0	804	0	NA	NA	648	1	57	MALE	NA	T2a	N0	M0	IS2
TCGA-KK-A8IG	2505	0	2505	0	2505	0	2505	0	55	MALE	NA	T2a	N0	M0	IS3
TCGA-KK-A8IH	2013	0	2013	0	2013	0	2013	0	50	MALE	NA	T2b	N0	M0	IS2
TCGA-KK-A8II	3467	1	3467	1	NA	NA	626	1	61	MALE	NA	T2c	N0	M0	IS2
TCGA-KK-A8IJ	1559	0	1559	0	NA	NA	307	1	59	MALE	NA	T3a	N1	M0	IS4
TCGA-KK-A8IK	2052	0	2052	0	2052	0	2052	0	56	MALE	NA	T1c	N0	M0	IS2
TCGA-KK-A8IL	628	1	628	NA	NA	NA	628	0	65	MALE	NA	T1c	N1	M0	IS1
TCGA-KK-A8IM	1915	0	1915	0	1915	0	1915	0	55	MALE	NA	T2a	N0	M0	IS1

TCGA-M7-A71Y	493	0	493	0	493	0	493	0	55	MALE	NA	T1c			IS4
TCGA-M7-A71Z	643	0	643	0	643	0	643	0	62	MALE	NA	T2a	N0		IS2
TCGA-M7-A720	384	0	384	0	384	0	384	0	53	MALE	NA	T1c			IS4
TCGA-M7-A721	400	0	400	0	400	0	400	0	70	MALE	NA	T1c		M0	IS4
TCGA-M7-A722	1060	0	1060	0	NA	NA	559	1	65	MALE	NA	T2a	N0	M0	IS2
TCGA-M7-A723	763	0	763	0	763	0	763	0	54	MALE	NA	T1c	N1	M0	IS1
TCGA-M7-A724	889	0	889	0	NA	NA	889	0	64	MALE	NA	T1c	N0	M0	IS2
TCGA-M7-A725	573	0	573	0	573	0	573	0	56	MALE	NA	T3a	N0	M0	IS2
TCGA-MG-AAMC	173	0	173	0	173	0	173	0	59	MALE	NA	T1c	N0	M0	IS2
TCGA-QU-A6IL	97	0	97	0	97	0	97	0	64	MALE	NA	T2a	N0	M0	IS2
TCGA-QU-A6IM	1247	0	1247	0	1247	0	1247	0	59	MALE	NA	T2b	N0	M0	IS1
TCGA-QU-A6IN	4264	0	4264	0	4264	0	4264	0	61	MALE	NA	T1c	N0	M0	IS2
TCGA-QU-A6IO	3716	0	3716	0	3716	0	3716	0	53	MALE	NA	T1c	N0	M0	IS2
TCGA-QU-A6IP	2620	0	2620	0	2620	0	2620	0	66	MALE	NA	T2a	N0	M0	IS4
TCGA-SU-A7E7	551	0	551	0	551	0	551	0	60	MALE	NA	T2b	N0	M0	IS1
TCGA-TK-A8OK	27	0	27	0	NA	NA	27	0	73	MALE	NA			M0	IS3
TCGA-TP-A8TT	526	0	526	0	NA	NA	526	0	64	MALE	NA	T1c	N0	M0	IS1
TCGA-TP-A8TV	610	0	610	0	610	0	610	0	62	MALE	NA	T1c	N0	M0	IS2
TCGA-V1-A8MF	1449	0	1449	0	1449	0	1449	0	65	MALE	NA	T2c	N0	M0	IS1
TCGA-V1-A8MG	857	0	857	0	857	0	857	0	53	MALE	NA	T2c		M0	IS1
TCGA-V1-A8MK	508	0	508	0	508	0	508	0	41	MALE	NA	T2a	N0	M0	IS4
TCGA-V1-A8ML	448	0	448	0	448	0	448	0	63	MALE	NA	T2b	N0	M0	IS4
TCGA-V1-A8MM	1195	0	1195	0	990	1	990	1	60	MALE	NA	T2b	N0	M0	IS1
TCGA-V1-A8MU	1876	0	1876	0	NA	NA	1876	0	56	MALE	NA	T2b	N1	M0	IS1
TCGA-V1-A8WL	1948	0	1948	0	1948	0	1948	0	64	MALE	NA	T1c	N0	M0	IS4

TCGA-V1-A8WN	924	0	924	0	924	0	924	0	47	MALE	NA	T2a		M0	IS4
TCGA-V1-A8WS	474	0	474	0	NA	NA	474	0	56	MALE	NA	T1c		M0	IS2
TCGA-V1-A8WV	686	0	686	0	686	0	686	0	52	MALE	NA	T2a	N1	M0	IS1
TCGA-V1-A8WW	1078	0	1078	0	1078	0	1078	0	59	MALE	NA	T2b	N1	M0	IS2
TCGA-V1-A8X3	202	0	202	0	202	0	202	0	51	MALE	NA	T1c	N0	M0	IS3
TCGA-V1-A9O5	1932	0	1932	0	124	1	124	1	64	MALE	NA	T2b	N1	M0	IS1
TCGA-V1-A9O7	2499	0	2499	0	NA	NA	819	1	60	MALE	NA	T2b	N0	M0	IS3
TCGA-V1-A9O9	2870	0	2870	0	2870	0	2870	0	56	MALE	NA	T3b	N0	M0	IS1
TCGA-V1-A9OA	636	0	636	0	636	0	636	0	61	MALE	NA	T2c	N1	M0	IS3
TCGA-V1-A9OF	1184	0	1184	0	1184	0	1184	0	49	MALE	NA	T1c		M0	IS4
TCGA-V1-A9OH	2403	0	2403	0	2403	0	2403	0	63	MALE	NA	T1c		M0	IS3
TCGA-V1-A9OL	1913	0	1913	0	105	1	105	1	65	MALE	NA	T2	N0	M0	IS1
TCGA-V1-A9OQ	371	0	371	0	371	0	371	0	67	MALE	NA	T1c		M0	IS4
TCGA-V1-A9OT	476	0	476	0	NA	NA	292	1	61	MALE	NA	T1c		M0	IS2
TCGA-V1-A9OX	1134	0	1134	0	NA	NA	1134	0	56	MALE	NA	T2c	N0	M0	IS1
TCGA-V1-A9OY	1067	0	1067	0	1067	0	1067	0	57	MALE	NA	T2	N1	M0	IS1
TCGA-V1-A9Z7	874	0	874	0	874	0	874	0	54	MALE	NA	T2	N0	M0	IS3
TCGA-V1-A9Z8	827	0	827	0	827	0	827	0	59	MALE	NA	T3b	N0	M0	IS3
TCGA-V1-A9Z9	540	0	540	0	540	0	540	0	63	MALE	NA	T1c	N0	M0	IS3
TCGA-V1-A9ZG	1517	0	1517	0	1517	0	1517	0	64	MALE	NA	T1c	N0	M0	IS3
TCGA-V1-A9ZI	1640	0	1640	0	NA	NA	1640	0	68	MALE	NA	T3b	N0	M0	IS1
TCGA-V1-A9ZK	1383	0	1383	0	1383	0	1383	0	68	MALE	NA	T2a	N0	M0	IS4
TCGA-V1-A9ZR	1361	0	1361	0	1361	0	1361	0	68	MALE	NA	T2c	N0	M0	IS1
TCGA-VN-A88I	269	0	269	0	NA	NA	269	0	59	MALE	NA	T1c	N0	M0	IS3
TCGA-VN-A88K	776	0	776	0	776	0	776	0	58	MALE	NA	T1c	N1	M0	IS2

TCGA-VN-A88L	746	0	746	0	746	0	746	0	54	MALE	NA	T1c	N0	M0	IS4
TCGA-VN-A88M	189	0	189	0	189	0	189	0	55	MALE	NA	T1c	N0	M0	IS2
TCGA-VN-A88N	766	0	766	0	766	0	766	0	62	MALE	NA	T1c	N0	M0	IS2
TCGA-VN-A88O	481	0	481	0	481	0	481	0	49	MALE	NA	T1c	N0	M0	IS1
TCGA-VN-A88P	1260	0	1260	0	1260	0	1260	0	60	MALE	NA	T1c	N0	M0	IS4
TCGA-VN-A88Q	1366	0	1366	0	1366	0	1366	0	60	MALE	NA	T2a	N0	M0	IS2
TCGA-VN-A88R	784	0	784	0	NA	NA	512	1	53	MALE	NA	T1c	N0	M0	IS2
TCGA-VN-A943	496	0	496	0	496	0	496	0	71	MALE	NA	T1c	N0	M0	IS4
TCGA-VP-A872	3631	0	3631	0	3631	0	3631	0	60	MALE	NA	T2	N0	M0	IS1
TCGA-VP-A875	2309	0	2309	0	2309	0	2309	0	67	MALE	NA	T2a	N0	M0	IS2
TCGA-VP-A876	3333	0	3333	0	3333	0	3333	0	46	MALE	NA	T1c	N0		IS2
TCGA-VP-A878	3130	0	3130	0	NA	NA	98	1	58	MALE	NA	T1c	N1		IS3
TCGA-VP-A879	728	1	728	0	728	0	728	0	70	MALE	NA	T1c	N0	M0	IS4
TCGA-VP-A87B	2722	0	2722	0	2722	0	2473	1	63	MALE	NA	T1c	N0	M0	IS2
TCGA-VP-A87C	1690	0	1690	0	1690	0	1690	0	67	MALE	NA	T1c	N0	M0	IS1
TCGA-VP-A87D	1853	0	1853	0	1194	1	1194	1	54	MALE	NA	T1c	N1	M0	IS2
TCGA-VP-A87E	2037	0	2037	0	2037	0	2037	0	59	MALE	NA	T1c	N0	M0	IS1
TCGA-VP-A87H	663	0	663	0	663	0	663	0	76	MALE	NA	T2	N0	M0	IS2
TCGA-VP-A87J	1774	0	1774	0	1774	0	1774	0	56	MALE	NA	T2a	N1	M0	IS2
TCGA-VP-A87K	1306	0	1306	0	533	1	533	1	63	MALE	NA	T1c	N0	M0	IS2
TCGA-VP-AA1N	1365	0	1365	0	1365	0	1365	0	70	MALE	NA	T1c	N0	M0	IS1
TCGA-WW-A8ZI	449	0	449	0	449	0	449	0	70	MALE	NA	T2c	N0	M0	IS1
TCGA-X4-A8KQ	1383	0	1383	0	NA	NA	1383	0	65	MALE	NA	T1c	N0	M0	IS1
TCGA-X4-A8KS	979	0	979	0	NA	NA	979	0	61	MALE	NA	T2c		M0	IS1
TCGA-XA-A8JR	376	0	376	0	376	0	376	0	68	MALE	NA	T2c	N0	M0	IS4

TCGA-XJ-A83F	1127	0	1127	0	1127	0	1127	0	67	MALE	NA	T3a	N0		IS2
TCGA-XJ-A83G	1461	0	1461	0	1461	0	1461	0	51	MALE	NA	T1c	N0		IS2
TCGA-XJ-A83H	1280	0	1280	0	NA	NA	1280	0	57	MALE	NA	T2a		M0	IS4
TCGA-XJ-A9DI	1461	0	1461	0	1461	0	1461	0	62	MALE	NA	T2b	N0	M0	IS1
TCGA-XJ-A9DK	275	0	275	0	275	0	275	0	63	MALE	NA	T1c		M0	IS4
TCGA-XJ-A9DQ	91	0	91	0	NA	NA	91	0	48	MALE	NA	T1c		M0	IS4
TCGA-XJ-A9DX	973	0	973	0	NA	NA	973	0	51	MALE	NA	T1c	N1	M1c	IS2
TCGA-XK-AAIR	972	0	972	0	972	0	972	0	75	MALE	NA	T1c	N0	M0	IS3
TCGA-XK-AAIV	1133	0	1133	0	1133	0	1133	0	63	MALE	NA	T1c	N1	M0	IS1
TCGA-XK-AAIW	1218	0	1218	0	1218	0	427	1	78	MALE	NA	T2b	N0	M0	IS1
TCGA-XK-AAJ3	1262	0	1262	0	1262	0	1262	0	56	MALE	NA	T1c	N0	M0	IS2
TCGA-XK-AAJA	930	0	930	0	930	0	930	0	62	MALE	NA	T2a	N0	M0	IS2
TCGA-XK-AAJP	1259	0	1259	0	1259	0	1259	0	66	MALE	NA	T1c	N0	M0	IS1
TCGA-XK-AAJR	1124	0	1124	0	131	1	131	1	61	MALE	NA	T1c	N0	M0	IS3
TCGA-XK-AAJT	1434	0	1434	0	NA	NA	1434	0	75	MALE	NA	T1c	N0	M0	IS3
TCGA-XK-AAJU	1576	0	1576	0	1576	0	1576	0	65	MALE	NA	T1c	N0	M0	IS4
TCGA-XK-AAK1	979	0	979	0	979	0	979	0	62	MALE	NA	T1c	N0	M0	IS2
TCGA-XQ-A8TA	146	1	146	1	NA	NA	146	1	59	MALE	NA	T3a		M1b	IS2
TCGA-XQ-A8TB	770	0	770	0	770	0	770	0	67	MALE	NA	T2a	N0	M0	IS3
TCGA-Y6-A8TL	992	0	992	0	NA	NA	992	0	65	MALE	NA	T2a	N0		IS4
TCGA-Y6-A9XI	526	0	526	0	526	0	526	0	72	MALE	NA		N0		IS2
TCGA-YJ-A8SW	148	0	148	0	NA	NA	148	0	67	MALE	NA	T1c	N0		IS2
TCGA-YL-A8HJ	1611	0	1611	0	1611	0	1611	0	58	MALE	NA	T3b		M0	IS3
TCGA-YL-A8HK	1491	0	1491	0	1376	1	1376	1	59	MALE	NA	T3b	N0	M0	IS1
TCGA-YL-A8HL	1565	0	1565	0	NA	NA	1565	0	58	MALE	NA	T3b	N1	M0	IS2

TCGA-YL-A8HM	1482	0	1482	0	1473	1	1473	1	66	MALE	NA	T3b	N0	M0	IS1
TCGA-YL-A8HO	2304	0	2304	0	NA	NA	1068	1	67	MALE	NA	T3a	N0	M0	IS1
TCGA-YL-A8S8	1890	0	1890	0	NA	NA	679	1	68	MALE	NA	T3a	N0	M0	IS2
TCGA-YL-A8S9	1842	0	1842	0	NA	NA	1006	1	63	MALE	NA	T3a	N0	M0	IS2
TCGA-YL-A8SA	512	0	512	0	NA	NA	512	0	69	MALE	NA	T2b	N0	M0	IS2
TCGA-YL-A8SB	1719	0	1719	0	NA	NA	1384	1	62	MALE	NA	T3a	N0	M0	IS3
TCGA-YL-A8SC	1261	0	1261	0	NA	NA	152	1	66	MALE	NA	T3a	N0	M0	IS1
TCGA-YL-A8SH	875	1	875	0	875	0	875	0	69	MALE	NA	T3a	N0	M0	IS2
TCGA-YL-A8SI	1614	0	1614	0	1423	1	1423	1	69	MALE	NA	T3a	N0	M0	IS3
TCGA-YL-A8SJ	1395	0	1395	0	752	1	752	1	60	MALE	NA	T3b	N0	M0	IS3
TCGA-YL-A8SK	1408	0	1408	0	NA	NA	1408	0	67	MALE	NA	T3b	N1	M0	IS1
TCGA-YL-A8SL	987	0	987	0	987	0	987	0	74	MALE	NA	T3b	N1	M0	IS2
TCGA-YL-A8SO	3479	0	3479	0	NA	NA	3479	0	64	MALE	NA	T1a		M0	IS2
TCGA-YL-A8SP	2366	0	2366	0	NA	NA	2036	1	58	MALE	NA	T2a		M0	IS2
TCGA-YL-A8SQ	2083	0	2083	0	NA	NA	329	1	61	MALE	NA	T3b	N0	M0	IS1
TCGA-YL-A8SR	273	0	273	0	NA	NA	273	0	64	MALE	NA	T1c	N0	M0	IS4
TCGA-YL-A9WH	427	0	427	0	NA	NA	427	0	67	MALE	NA	T3b	N1	M0	IS2
TCGA-YL-A9WI	1623	0	1623	0	NA	NA	1623	0	63	MALE	NA	T3a	N1	M0	IS2
TCGA-YL-A9WJ	1855	1	1855	1	NA	NA	1476	1	47	MALE	NA	T4	N1	M0	IS1
TCGA-YL-A9WK	2136	0	2136	0	NA	NA	1009	1	63	MALE	NA	T2b	N0	M0	IS1
TCGA-YL-A9WL	740	0	740	0	740	1	740	1	59	MALE	NA	T2a	N0	M0	IS2
TCGA-YL-A9WX	1518	0	1518	0	NA	NA	1506	1	68	MALE	NA	T3a	N1	M0	IS1
TCGA-YL-A9WY	1098	0	1098	0	NA	NA	765	1	57	MALE	NA	T3a	N1	M0	IS1
TCGA-ZG-A8QW	94	0	94	0	NA	NA	94	0	72	MALE	NA	T3a	N1	M0	IS1
TCGA-ZG-A8QX	442	0	442	0	NA	NA	442	0	56	MALE	NA	T1c	N0	M0	IS4

TCGA-ZG-A8QY	404	0	404	0	NA	NA	404	0	67	MALE	NA	T2c	N1	M0	IS3
TCGA-ZG-A8QZ	308	0	308	0	NA	NA	308	0	65	MALE	NA	T2a	N1	M0	IS1
TCGA-ZG-A9KY	130	0	130	0	NA	NA	130	0	73	MALE	NA	T1c	N1	M0	IS1
TCGA-ZG-A9L0	355	0	355	0	NA	NA	355	0	71	MALE	NA	T3b	N1	M0	IS1
TCGA-ZG-A9L1	1302	0	1302	0	NA	NA	1302	0	66	MALE	NA	T1c	N1	M0	IS2
TCGA-ZG-A9L2	1154	0	1154	0	NA	NA	180	1	70	MALE	NA	T3a	N0	M0	IS3
TCGA-ZG-A9L4	1108	0	1108	0	NA	NA	1108	0	61	MALE	NA	T1c	N0	M0	IS3
TCGA-ZG-A9L5	908	0	908	0	NA	NA	908	0	58	MALE	NA	T2a	N1	M0	IS2
TCGA-ZG-A9L6	687	0	687	0	NA	NA	664	1	64	MALE	NA	T1c	N0	M0	IS3
TCGA-ZG-A9L9	617	0	617	0	NA	NA	51	1	60	MALE	NA	T2b	N1	M0	IS1
TCGA-ZG-A9LB	583	0	583	0	NA	NA	583	0	72	MALE	NA	T2a	N0	M0	IS1
TCGA-ZG-A9LM	378	0	378	0	378	0	378	0	72	MALE	NA	T2b	N1	M0	IS1
TCGA-ZG-A9LN	115	0	115	0	NA	NA	115	0	57	MALE	NA	T1c	N1	M0	IS1
TCGA-ZG-A9LS	516	0	516	0	516	0	516	0	64	MALE	NA	T2a	N1	M0	IS1
TCGA-ZG-A9LU	579	0	579	0	NA	NA	579	0	67	MALE	NA	T2b	N0	M0	IS1
TCGA-ZG-A9LY	370	0	370	0	370	0	370	0	60	MALE	NA	T2b	N1	M0	IS3
TCGA-ZG-A9LZ	692	0	692	0	NA	NA	692	0	66	MALE	NA	T3a	N0	M0	IS1
TCGA-ZG-A9M4	547	0	547	0	NA	NA	547	0	65	MALE	NA	T2a	N1	M0	IS1
TCGA-ZG-A9MC	455	0	455	0	NA	NA	455	0	69	MALE	NA	T2a	N1	M0	IS1
TCGA-ZG-A9N3	349	0	349	0	NA	NA	349	0	73	MALE	NA	T1c	N1	M0	IS1
TCGA-ZG-A9ND	410	0	410	0	410	0	410	0	55	MALE	NA	T1c	N0	M0	IS2
TCGA-ZG-A9NI	132	0	132	0	132	0	132	0	73	MALE	NA	T2a	N0	M0	IS3
