# Supplementary material for: Development of a Novel Immune Subtyping System Expanded with Immune Landscape and an 11-Gene Signature for Predicting Prostate Cancer Survival
Source: J Oncol. 2022 Feb 16;2022:1183173. doi: 10.1155/2022/1183173 (PMC8866019; doi:10.1155/2022/1183173)
Supplement: Supplementary Materials — Supplementary Figure S1. Work flow chart. Supplementary Figure S2. Expression and interaction analysis of 11 genes. (A) Differential expression of 11 genes in cancer and adjacent tissues. (B) Correlation between 11 genes and immune infiltrating cells. (C) Correlation analysis between the expression of 11 genes and immune checkpoint genes. (D) Interaction between 11 genes. Supplementary Table S1. Immune related genes list. Supplementary Table S2. Clinical information of TCGA-PRAD dataset. Supplementary Table S3. Clinical information of MKSCC-PRAD dataset. Supplementary Table S4. Information of co-expression module corresponding to each gene. [file 1183173.f1.zip › 1183173.f1/Supplementary Table S3.pdf]

Supplementary Table S3. Clinical information of MKSCC-PRAD dataset

Sample	DFS.time	DFS	Gender
PCA0038	3006.3	0	Male
PCA0084	1189.8	0	Male
PCA0150	1540.5	0	Male
PCA0058	905.7	0	Male
PCA0210	601.2	1	Male
PCA0215	178.5	1	Male
PCA0080	1661.7	1	Male
PCA0035	2334	0	Male
PCA0081	295.8	1	Male
PCA0082	1769.1	0	Male
PCA0036	2559.6	0	Male
PCA0037	3121.5	0	Male
PCA0083	954	1	Male
PCA0040	2685	0	Male
PCA0086	1825.5	0	Male
PCA0087	1705.2	0	Male
PCA0089	2105.4	0	Male
PCA0090	1452.9	0	Male
PCA0092	504.6	1	Male
PCA0093	1392.6	0	Male
PCA0094	1983	0	Male
PCA0095	1547.4	0	Male
PCA0096	1271.4	0	Male
PCA0097	56.1	0	Male
PCA0099	173.4	0	Male
PCA0100	1146.3	0	Male
PCA0101	1870.8	0	Male
PCA0103	859.5	1	Male
PCA0104	1304.1	0	Male
PCA0105	690	0	Male
PCA0107	1684.5	0	Male
PCA0108	1774.2	0	Male
PCA0109	415.8	0	Male
PCA0110	1767.3	0	Male
PCA0111	1495.2	0	Male
PCA0187	540	1	Male
PCA0112	396.3	1	Male
PCA0113	1813.5	0	Male
PCA0114	1163.1	0	Male
PCA0115	786.6	0	Male
PCA0117	564.9	1	Male

PCA0118	1314.9	0	Male
PCA0119	1394.7	0	Male
PCA0120	1879.5	0	Male
PCA0122	1574.1	0	Male
PCA0123	1801.8	0	Male
PCA0124	1656	0	Male
PCA0125	1841.1	0	Male
PCA0126	1555.5	0	Male
PCA0127	1305.9	0	Male
PCA0128	1614.6	1	Male
PCA0129	1706.1	0	Male
PCA0130	835.8	1	Male
PCA0132	1456.8	0	Male
PCA0133	841.8	0	Male
PCA0134	245.4	0	Male
PCA0135	1549.5	0	Male
PCA0136	171.6	1	Male
PCA0141	1252.8	0	Male
PCA0144	1127.7	0	Male
PCA0145	1452.9	0	Male
PCA0146	1526.7	0	Male
PCA0147	1371.9	0	Male
PCA0149	1775.1	0	Male
PCA0151	1420.2	0	Male
PCA0155	1428.3	0	Male
PCA0156	324.3	0	Male
PCA0157	1195.5	0	Male
PCA0158	948.3	0	Male
PCA0159	42.3	1	Male
PCA0160	389.4	0	Male
PCA0050	2515.5	0	Male
PCA0161	570.6	1	Male
PCA0162	354.9	0	Male
PCA0163	1361.1	0	Male
PCA0164	1636.2	0	Male
PCA0165	915.6	0	Male
PCA0167	805.2	0	Male
PCA0168	1470.6	0	Male
PCA0169	1288.2	0	Male
PCA0170	1565.1	0	Male
PCA0171	265.2	0	Male
PCA0172	916.8	1	Male
PCA0173	980.7	0	Male
PCA0174	1299	0	Male

PCA0175	1080.3	0	Male
PCA0052	3076.2	0	Male
PCA0054	63	1	Male
PCA0176	76.8	1	Male
PCA0056	750	0	Male
PCA0057	2485.8	0	Male
PCA0062	1288.2	0	Male
PCA0063	2223.6	0	Male
PCA0064	728.4	0	Male
PCA0178	1130.4	0	Male
PCA0200	86.7	1	Male
PCA0065	2321.1	0	Male
PCA0201	123.3	1	Male
PCA0066	2313.3	0	Male
PCA0179	87.6	1	Male
PCA0206	48.3	1	Male
PCA0180	41.4	1	Male
PCA0207	41.4	1	Male
PCA0181	900.9	0	Male
PCA0005	3783	0	Male
PCA0208	353.7	1	Male
PCA0213	1939.8	1	Male
PCA0068	1366.2	0	Male
PCA0073	391.2	1	Male
PCA0074	2529.3	0	Male
PCA0075	1480.5	0	Male
PCA0077	1851	0	Male
PCA0001	555	1	Male
PCA0007	2958	0	Male
PCA0008	4475.7	0	Male
PCA0009	1942.8	1	Male
PCA0010	1051.8	0	Male
PCA0011	2465.1	0	Male
PCA0012	3852.9	0	Male
PCA0013	311.4	0	Male
PCA0002	1740.6	0	Male
PCA0014	2293.5	0	Male
PCA0015	681	0	Male
PCA0017	3131.4	0	Male
PCA0018	568.8	0	Male
PCA0020	1708.2	0	Male
PCA0021	1845	0	Male
PCA0022	1198.5	1	Male
PCA0024	118.2	1	Male

PCA0025	2041.2	1	Male
PCA0003	2794.2	0	Male
PCA0026	2344.8	0	Male
PCA0027	3504.9	0	Male
PCA0028	828	1	Male
PCA0029	800.4	0	Male
PCA0030	3452.7	0	Male
PCA0031	1060.5	1	Male
PCA0032	111.3	1	Male
PCA0033	2072.7	0	Male
PCA0034	2789.4	1	Male
