# Supplementary material for: Development of a Novel Immune Subtyping System Expanded with Immune Landscape and an 11-Gene Signature for Predicting Prostate Cancer Survival
Source: J Oncol. 2022 Feb 16;2022:1183173. doi: 10.1155/2022/1183173 (PMC8866019; doi:10.1155/2022/1183173)
Supplement: Supplementary Materials — Supplementary Figure S1. Work flow chart. Supplementary Figure S2. Expression and interaction analysis of 11 genes. (A) Differential expression of 11 genes in cancer and adjacent tissues. (B) Correlation between 11 genes and immune infiltrating cells. (C) Correlation analysis between the expression of 11 genes and immune checkpoint genes. (D) Interaction between 11 genes. Supplementary Table S1. Immune related genes list. Supplementary Table S2. Clinical information of TCGA-PRAD dataset. Supplementary Table S3. Clinical information of MKSCC-PRAD dataset. Supplementary Table S4. Information of co-expression module corresponding to each gene. [file 1183173.f1.zip › 1183173.f1/Supplementary Table S4.pdf]

Supplementary Table S4. Information of co-expression module corresponding to each gene

Tag	mergedColors
A2M	black
ABCA6	black
ABCA8	black
ABCA9	black
ABCB1	black
ABCC9	black
ABCD2	blue
ABI3	blue
ABI3BP	blue
ACAP1	blue
ACE	grey
ACHE	grey
ACOXL	grey
ACP5	grey
ACSL5	grey
ACSM5	blue
ACSS3	black
ACTA2	black
ACTN1	black
ACVR1	turquoise
ACVR1B	turquoise
ACVR2A	turquoise
ACVR2B	turquoise
ACVRL1	black
ADAM12	blue
ADAM28	blue
ADAM8	blue
ADAMDEC1	blue
ADAMTS10	black
ADAMTS12	black
ADAMTS14	blue
ADAMTS16	grey
ADAMTS2	black
ADAMTS4	grey
ADAMTS5	black
ADAMTS9	black
ADAMTSL2	black
ADAP2	blue
ADAT1	turquoise
ADCY4	black
ADCYAP1	grey
ADORA2A	grey

ADPRH	grey
ADRA2A	grey
ADRM1	grey
AEBP1	black
AFF3	turquoise
AGAP2	blue
AGTR1	grey
AHCYL2	turquoise
AIF1	blue
CRYBG2	grey
AIM2	blue
AKAP12	black
AKAP2	turquoise
AKAP5	turquoise
AKNA	blue
ALDH1A1	grey
ALDH3B1	blue
ALOX5	grey
ALOX5AP	blue
ALOXE3	grey
ALPK2	grey
AMH	grey
AMHR2	grey
JAML	blue
AMIGO3	grey
AMPD1	grey
AMPH	black
ANGPTL1	black
ANGPTL2	black
ANK2	black
ANKRD17	turquoise
ANKRD22	grey
ANKRD44	blue
ANKRD55	blue
SOWAHD	blue
ANO6	black
ANTXR1	black
ANTXR2	black
ANXA6	black
AOAH	blue
AOC3	black
AP1S2	blue
AP3B1	turquoise
APBB1IP	blue

APBB2	turquoise
APLNR	black
APOBR	blue
APOBEC3A	grey
APOBEC3D	blue
APOBEC3G	blue
APOBEC3H	blue
APOC1	blue
APOC2	grey
APOE	blue
APOL3	blue
APOL6	greenyellow
AQP10	grey
AQP1	black
AQP9	blue
AREG	grey
ARHGAP15	blue
ARHGAP18	turquoise
ARHGAP22	blue
ARHGAP25	blue
ARHGAP30	blue
ARHGAP31	black
ARHGAP4	blue
ARHGAP6	turquoise
ARHGAP9	blue
ARHGDIB	blue
ARHGEF15	black
ARHGEF37	turquoise
ARHGEF6	blue
ARID5A	blue
ARL6IP5	turquoise
ARRB1	grey
ARRB2	blue
ARRDC5	blue
ARSB	turquoise
ART4	grey
ASAH1	turquoise
CLMP	black
ASGR2	blue
ASPN	black
ASRGL1	grey
ASXL2	turquoise
ASXL3	black
ATE1	turquoise

ATP10A	turquoise
ATP2A3	grey
ATP8A1	turquoise
ATP8B4	grey
AVPR1A	black
AZGP1	grey
B2M	blue
B3GAT1	grey
BANK1	grey
BATF	blue
BATF2	blue
BATF3	blue
BCL2A1	blue
BCL2L14	grey
BCL6B	black
BDKRB2	black
BEND5	black
BEX5	grey
BFSP2	blue
BGN	black
BHLHA15	turquoise
BHLHE22	blue
BHLHE41	grey
BICC1	turquoise
BIN2	blue
BIRC6	turquoise
BLK	blue
BMP2	grey
BMP2K	turquoise
BMP7	grey
BMPR1A	turquoise
BMPR1B	turquoise
BMPR2	turquoise
BNC2	black
BST1	black
BST2	blue
BTK	blue
BTLA	blue
BTN2A2	blue
BTN3A1	greenyellow
BTN3A2	blue
BTN3A3	greenyellow
VSIR	blue
VSTM4	black

C10orf99	grey
C11orf21	blue
RUBCNL	blue
MEDAG	grey
CEP128	turquoise
SLIRP	turquoise
RTRAF	grey
C15orf48	grey
C16orf54	blue
MILR1	blue
SCIMP	blue
LDLRAD4	black
PEAK3	blue
C19orf38	blue
TRIR	turquoise
MCEMP1	grey
C1orf116	turquoise
C1orf127	grey
GCSAML	grey
C1orf162	blue
PIK3CD-AS1	blue
THEMIS2	blue
C1orf54	black
C1QA	blue
C1QB	blue
C1QC	blue
C1QTNF7	black
LAMP5	grey
C2	grey
RTP5	blue
TRABD2A	grey
C3	blue
C3AR1	blue
C4A	grey
NDNF	black
C5AR1	blue
CREBRF	turquoise
C5orf56	blue
ADTRP	grey
UQCC2	turquoise
C6orf132	turquoise
CCDC170	grey
C7	black
CPED1	black

NUGGC	blue
ERCC6L2	turquoise
C9orf139	grey
CYSRT1	grey
CACNA1C	black
CACNA2D2	turquoise
CACNA2D4	blue
CALB2	grey
CALD1	black
CALR	turquoise
CAMK1	grey
CAMK4	black
CANX	turquoise
CARD11	blue
CARD8	turquoise
CARD9	blue
CASP5	blue
CASS4	blue
CAV1	black
CCDC102B	grey
CCDC141	grey
CCDC69	black
CCDC80	black
CCL11	grey
CCL13	blue
CCL14	black
CCL15	grey
CCL16	grey
CCL17	blue
CCL18	blue
CCL19	blue
CCL20	blue
CCL21	black
CCL22	blue
CCL23	blue
CCL24	grey
CCL25	grey
CCL26	grey
CCL2	blue
CCL28	blue
CCL3	grey
CCL3L1	grey
CCL3L3	grey
CCL4	blue

CCL4L1	grey
CCL5	blue
CCL7	grey
CCL8	grey
CCNT1	turquoise
CCR10	grey
CCR1	blue
CCR2	blue
CCR3	blue
CCR4	blue
CCR5	blue
CCR6	blue
CCR7	blue
CCR8	blue
CCR9	blue
CCRL2	blue
CD14	blue
CD160	blue
CD163	blue
CD163L1	grey
CD180	blue
CD19	blue
CD1A	blue
CD1B	blue
CD1C	blue
CD1D	blue
CD1E	blue
CD200	black
CD200R1	blue
CD207	blue
CD209	blue
CD226	blue
CD22	blue
CD244	blue
CD247	blue
CD248	black
CD274	blue
CD27	blue
CD28	blue
CD2	blue
CD300A	blue
CD300C	blue
CD300E	blue
CD300LB	blue

CD300LF	blue
CD302	turquoise
CD33	blue
CD34	black
CD36	grey
CD37	blue
CD3D	blue
CD3E	blue
CD3G	blue
CD40	blue
CD40LG	blue
CD48	blue
CD4	blue
CD52	blue
CD53	blue
CD58	grey
CD5	blue
CD68	blue
CD6	blue
CD69	blue
CD70	blue
CD72	blue
CD74	blue
CD7	blue
CD79A	blue
CD79B	blue
CD80	blue
CD84	blue
CD86	blue
CD8A	blue
CD8B	blue
CD93	black
CD96	blue
ADGRE5	blue
CDC42SE2	turquoise
CDH11	black
CDH20	grey
CDH3	grey
CDH5	black
CDH6	black
CDK15	black
CDKL5	turquoise
CEACAM19	grey
CEACAM21	blue

CEACAM4	blue
ADA2	blue
CELF2	black
CERKL	blue
CETP	grey
CFP	blue
CHAC1	grey
CHIT1	grey
CHN1	grey
CHRD	grey
CHRDL1	black
CHRNA6	blue
CHST13	blue
CHST2	black
CIITA	blue
CILP	grey
CISH	grey
CLCF1	grey
CLEC10A	blue
CLEC11A	grey
CLEC12A	blue
CLEC14A	black
CLEC1A	black
CLEC3B	black
CLEC4A	blue
CLEC4D	blue
CLEC4E	blue
CLEC4G	grey
CLEC4M	grey
CLEC5A	blue
CLEC9A	blue
CLECL1	blue
CLIC2	blue
CLIC3	black
CLIC5	black
CLIP3	black
CLNK	grey
CLOCK	turquoise
CLTB	turquoise
CMA1	grey
CMAHP	blue
CMKLR1	blue
CMPK2	greenyellow
CNFN	turquoise

CNR2	blue
CNRIP1	black
CNTF	grey
CNTFR	black
COL10A1	black
COL11A1	black
COL12A1	grey
COL14A1	black
COL15A1	black
COL18A1	black
COL1A1	black
COL1A2	black
COL3A1	black
COL4A1	black
COL4A2	black
COL5A1	black
COL5A2	black
COL5A3	black
COL6A1	black
COL6A2	black
COL6A3	black
COL6A6	grey
COL8A1	black
COLEC12	grey
CORIN	grey
CORO1A	blue
COTL1	grey
CPA3	black
CPNE5	blue
CPVL	blue
CPXM1	black
CPZ	black
CR1	blue
CR1L	blue
CR2	blue
CREB1	turquoise
CREB3L1	grey
CREBL2	turquoise
CRISPLD2	black
CRLF2	grey
CRTAM	blue
CRYBB1	grey
CSF1	blue
CSF1R	blue

CSF2RA	blue
CSF2RB	blue
CSF3	grey
CSF3R	blue
CSGALNACT2	turquoise
CSMD2	grey
CST7	blue
CTF1	black
CTHRC1	black
CTLA4	blue
CTSB	blue
CTSE	grey
CTSG	grey
CTSK	black
CTSL	turquoise
CTSO	turquoise
CTSS	blue
CTSW	blue
CTSZ	blue
CTTNBP2	black
CX3CL1	blue
CX3CR1	blue
CXCL10	blue
CXCL11	blue
CXCL12	black
CXCL1	blue
CXCL13	blue
CXCL16	blue
CXCL2	grey
CXCL3	grey
CXCL5	grey
CXCL6	blue
CXCL9	blue
CXCR1	grey
CXCR2	grey
CXCR3	blue
CXCR4	blue
CXCR5	blue
CXCR6	blue
ACKR3	black
CXorf21	blue
CXorf65	blue
CYBA	blue
CYBB	blue

CYFIP2	turquoise
CYP1B1	grey
CYP27A1	black
CYSLTR1	blue
CYSLTR2	blue
CYTH4	blue
CYTIP	blue
DAAM2	black
DAB2	black
DACT1	black
DACT3	black
ACKR1	black
DBH	grey
DCBLD1	turquoise
DCHS1	black
DCN	black
DDI2	turquoise
DDR2	black
DDX58	greenyellow
DDX60	greenyellow
DENND1C	turquoise
DENND2A	black
DERL3	grey
DHRS1	grey
DHRS9	grey
DIXDC1	black
DKK2	black
DLC1	black
DLL4	black
DMKN	black
DMXL2	turquoise
DNAH8	grey
DNAJC5B	blue
DOCK10	blue
DOCK11	blue
DOCK2	blue
DOCK4	turquoise
DOCK8	blue
DOK1	blue
DOK2	blue
DOK3	blue
DOK5	black
DOK6	turquoise
DPEP1	grey

DPEP2	blue
DPP8	turquoise
DPT	black
DSC1	grey
DSG1	grey
DSP	turquoise
DUOXA1	grey
DUSP16	turquoise
DUSP4	grey
DYSF	black
E2F5	turquoise
EBF1	black
EBF2	grey
EBI3	blue
ECM2	black
ECSCR	black
EDA	grey
EDA2R	black
EDAR	grey
EDNRA	black
EDNRB	black
EFEMP2	black
EFTUD2	turquoise
EGF	grey
EGFR	turquoise
EHD2	black
ADGRL4	black
EMCN	black
EMILIN1	black
EMILIN2	grey
ADGRE2	blue
ADGRE4P	blue
ENG	black
ENO3	grey
ENOX1	grey
ENPEP	black
ENPP2	black
ENPP3	grey
ENPP4	turquoise
ENTPD1	black
EOMES	blue
EP300	turquoise
EPCAM	turquoise
EPO	grey

EPOR	grey
EPS8	black
EPS8L1	grey
EPSTI1	blue
ERAP1	turquoise
ERAP2	grey
ERN1	turquoise
ERP27	grey
ESAM	black
ETS1	blue
ETV3	turquoise
ETV7	blue
EVI2A	blue
EVI2B	blue
EVPL	grey
F13A1	blue
F2R	black
F5	turquoise
FABP3	black
FCMR	blue
FAM107A	black
PCED1B	blue
DENND6B	grey
FAM13C	turquoise
FAM155A	grey
FAM168A	turquoise
FAM171B	turquoise
FAM177B	blue
TVP23A	black
CCSER1	turquoise
CALHM5	black
CALHM6	blue
PIEZO2	black
STRIP2	grey
FAM49A	grey
NXPE4	grey
MINDY2	turquoise
RIPOR2	grey
FAM78A	blue
FAM83A	grey
FAM92B	grey
FAP	black
FAS	turquoise
FASLG	blue

FAT4	black
FBLN2	black
FBLN5	black
FBN1	black
FBP1	grey
FBXL7	black
FBXO6	grey
FCAR	grey
FCER1A	blue
FCER1G	blue
FCER2	blue
FCGBP	grey
FCGR1A	blue
FCGR1B	blue
FCGR1CP	grey
FCGR2A	blue
FCGR2B	blue
FCGR2C	blue
FCGR3A	blue
FCGR3B	grey
FCGRT	grey
FCN1	blue
FCRL1	blue
FCRL2	blue
FCRL3	blue
FCRL5	blue
FCRL6	blue
FCRLA	blue
FERMT2	black
FERMT3	blue
FGD2	blue
FGD3	blue
FGD5	black
FGF14	grey
FGF7	black
FGL2	blue
FGR	blue
FHL5	black
FIBIN	black
FICD	turquoise
VEGFD	grey
FILIP1L	black
FKBP11	grey
FKBP7	black

FLI1	blue
ANKRD36BP2	blue
FLT1	black
FLT3	blue
FLT3LG	blue
FLT4	black
FLVCR2	grey
FMNL1	blue
FMNL3	blue
FMOD	grey
FN1	black
FNBP1	black
FNDC1	black
FNIP2	turquoise
FOLR2	blue
FOXP3	blue
FPR1	blue
FPR2	blue
FPR3	blue
FRZB	black
FSCN1	black
FSTL1	turquoise
FSTL3	black
FUCA1	turquoise
FUT7	blue
FYB1	blue
FYN	blue
FZD4	turquoise
GAB3	blue
GALM	grey
GALNT15	black
GAPT	blue
GAS7	black
GATA1	grey
GATA2	turquoise
GATA3	blue
GATM	black
GBGT1	blue
GBP1	blue
GBP2	blue
GBP4	blue
GBP5	blue
GCSAM	blue
GDF5	grey

GFI1	blue
GFRA3	grey
GGT1	grey
GGT5	black
GGTA1P	blue
GHR	turquoise
GHRL	grey
GIMAP1	blue
GIMAP2	blue
GIMAP4	blue
GIMAP5	blue
GIMAP6	blue
GIMAP7	blue
GIMAP8	blue
GIPC3	black
GIT2	turquoise
GJA4	black
GJA5	black
GJB2	grey
GJB3	grey
GJB5	grey
GJD3	grey
GLIPR2	grey
GLIS3	black
GLRX	grey
COLGALT2	black
GLT8D2	black
GMFG	blue
GMIP	blue
GMPR	grey
GNA15	grey
GNAI2	black
GNG11	black
GNG2	blue
GNG7	grey
GNGT2	blue
GNLY	blue
GNS	turquoise
GPBAR1	blue
GPC5	grey
GPC6	black
GPIHBP1	black
ADGRG5	blue
ADGRF4	grey

ADGRF5	turquoise
ADGRA2	black
GPR132	blue
ADGRD1	black
GPR137B	turquoise
GPR141	blue
GPR15	blue
GPR157	turquoise
GPR171	blue
GPR174	blue
GPR18	blue
GPR183	blue
GPR25	blue
GPR34	blue
GPR35	grey
GPR4	black
GPR55	blue
GPR65	blue
GPR78	grey
GPR82	blue
GPR84	blue
GPRIN3	blue
GPSM3	blue
GRAP	blue
GRAP2	blue
GREM1	black
GRIN3A	grey
GSDMA	grey
GTF2A1	turquoise
GUCY1A2	black
GVINP1	blue
GYPC	black
GZMA	blue
GZMB	blue
GZMH	blue
GZMK	blue
GZMM	blue
HAMP	blue
HAPLN3	blue
HAVCR1	grey
HAVCR2	blue
HCK	blue
HCLS1	blue
HCP5	blue

HCST	blue
HDC	black
HECW2	black
HEPH	black
HEPHL1	grey
HERC6	greenyellow
HERPUD1	grey
HEYL	black
HFE	black
HGF	black
HIC1	black
HIPK3	turquoise
HIST1H2AE	grey
HIST1H2AG	grey
HIST1H2AM	grey
HIST1H3H	grey
HK3	blue
HLA-A	blue
HLA-B	blue
HLA-C	blue
HLA-DMA	blue
HLA-DMB	grey
HLA-DOA	blue
HLA-DOB	blue
HLA-DPA1	blue
HLA-DPB1	blue
HLA-DPB2	blue
HLA-DQA1	blue
HLA-DQA2	blue
HLA-DQB1	blue
HLA-DQB2	blue
HLA-DRA	blue
HLA-DRB1	blue
HLA-DRB5	blue
HLA-DRB6	blue
HLA-E	blue
HLA-F	blue
HLA-G	grey
HLX	grey
HMCN1	black
ARHGAP45	blue
HMSD	blue
HNMT	grey
HPGD	grey

HPGDS	grey
HRH2	black
HS3ST1	grey
HS3ST2	grey
HSD11B1	black
HSD17B14	grey
HSH2D	grey
HSP90AA1	turquoise
HSP90AB1	turquoise
HSPA12B	black
HSPA1A	grey
HSPA1B	grey
HSPA1L	grey
HSPA2	grey
HSPA4	turquoise
HSPA5	turquoise
HSPA6	grey
HSPA8	turquoise
HTR2A	grey
HTRA3	black
HTRA4	blue
HVCN1	blue
HYDIN	grey
ICAM1	turquoise
ICAM2	black
ICAM3	blue
ICK	turquoise
ICOS	blue
ICOSLG	grey
IDO1	blue
IDO2	grey
IFFO1	black
IFI27	blue
IFI30	blue
IFI35	blue
IFI44	greenyellow
IFI44L	greenyellow
IFI6	greenyellow
IFIH1	greenyellow
IFIT2	greenyellow
IFIT3	greenyellow
IFIT5	greenyellow
IFITM1	blue
IFITM3	blue

IFNAR1	turquoise
IFNAR2	turquoise
IFNE	grey
IFNG	blue
IFNGR1	turquoise
IFNGR2	turquoise
IGDCC4	grey
IGF1	grey
JCHAIN	blue
IGLL1	blue
IGSF10	grey
IGSF21	grey
IGSF6	blue
IKZF1	blue
IKZF3	blue
IL10	blue
IL10RA	blue
IL10RB	grey
IL11	grey
IL11RA	grey
IL12A	blue
IL12B	blue
IL12RB1	blue
IL12RB2	blue
IL13	grey
IL13RA1	turquoise
IL15	blue
IL15RA	blue
IL16	blue
IL17B	black
IL17RA	turquoise
IL17RB	turquoise
IL18	blue
IL18BP	blue
IL18R1	blue
IL18RAP	blue
IL1A	grey
IL1B	blue
IL1R1	turquoise
IL1R2	blue
IL1RAP	turquoise
IL1RL1	grey
IL1RN	blue
IL20	grey

IL20RA	grey
IL20RB	grey
IL21R	blue
IL22RA1	grey
IL22RA2	blue
IL2	blue
IL23A	blue
IL23R	blue
IL24	blue
IL27	grey
IFNLR1	turquoise
IL2RA	blue
IL2RB	blue
IL2RG	blue
IL32	blue
IL3RA	blue
IL4	grey
IL4I1	blue
IL4R	blue
IL5	grey
IL5RA	grey
IL6	grey
IL6R	grey
IL6ST	turquoise
IL7	blue
IL7R	blue
CXCL8	grey
IL9R	blue
INHBA	black
INHBB	grey
INHBC	grey
INHBE	grey
INMT	black
INPP5D	blue
IPCEF1	blue
IQGAP2	turquoise
IRF1	blue
IRF4	blue
IRF7	grey
IRF8	blue
IRF9	greenyellow
ISG15	greenyellow
ISG20	grey
ISLR	black

ITGA11	black
ITGA1	black
ITGA2B	grey
ITGA4	blue
ITGA5	black
ITGA8	black
ITGA9	black
ITGAD	blue
ITGAL	blue
ITGAM	blue
ITGAX	blue
ITGB1	turquoise
ITGB2	blue
ITGB3	black
ITGB7	blue
ITGBL1	black
ITK	blue
ITM2A	black
IVL	grey
JAK2	turquoise
JAK3	blue
JAKMIP1	grey
JAM2	black
JAM3	black
JMY	turquoise
JSRP1	grey
JUP	turquoise
KCNA3	blue
KCNAB2	blue
KCND2	grey
KCNE4	black
KCNH2	black
KCNJ10	grey
KCNJ8	black
KCNK13	grey
KCNK6	grey
KCNMB1	black
KCNN3	black
KCNN4	grey
KCNT2	black
KCTD12	blue
KDR	black
FAM30A	blue
TESPA1	blue

KIAA0754	turquoise
JCAD	black
KIAA1549	turquoise
SHISAL1	black
KIAA1755	black
KIF21B	blue
KIR2DL4	grey
KIR3DL1	grey
KIR3DL2	grey
KIRREL1	black
KIT	black
KITLG	black
KL	grey
KLHDC10	turquoise
KLHL11	turquoise
KLHL23	turquoise
KLHL6	blue
KLK7	grey
KLRB1	blue
KLRC1	blue
KLRC2	grey
KLRC4	blue
KLRD1	blue
KLRG1	blue
KLRK1	blue
KMO	blue
KRT1	grey
KRT14	grey
KRT16	grey
KRT6A	grey
KRT6B	grey
KRT6C	grey
LAD1	grey
LAG3	blue
LAIR1	blue
LAIR2	blue
LAMA2	black
LAMA4	black
LAMC2	turquoise
LAP3	turquoise
LAPTM5	blue
LAT	blue
LAT2	grey
LATS1	turquoise

LATS2	turquoise
LAX1	blue
LCK	blue
LCN10	grey
LCOR	turquoise
LCP1	grey
LCP2	blue
LDB2	black
LEP	grey
LEPR	black
LGALS2	blue
LGALS9	blue
LGI2	blue
LGMN	turquoise
LHFPL6	black
LHFPL2	black
LIF	blue
LIFR	turquoise
LIG3	turquoise
LILRA1	blue
LILRA2	blue
LILRA4	blue
LILRA5	blue
LILRA6	blue
LILRB1	blue
LILRB2	blue
LILRB3	blue
LILRB4	blue
LILRB5	blue
LIMD2	blue
LIME1	grey
LIMS1	turquoise
LIPA	turquoise
LMOD1	black
LMTK2	turquoise
UNQ6494	grey
FAM83A-AS1	grey
LINC00426	blue
PCED1B-AS1	blue
LINC00654	black
LINC00926	blue
SMIM1	turquoise
LGALS17A	blue
LOC730101	grey

LOXL2	black
LOXL3	black
LPAR4	black
LPL	grey
PLPPR4	black
LPXN	grey
LRCH2	black
LRMP	blue
LRP6	turquoise
LRRC15	grey
LRRC17	black
LRRC25	blue
LRRC32	black
NRROS	blue
LSAMP	grey
LSP1	blue
LST1	blue
LTA	blue
LTB	blue
LTBP2	black
LTBR	grey
LTC4S	grey
LUM	black
LY86	blue
LY9	blue
LY96	blue
LYL1	blue
LYN	blue
LYPD3	grey
LYPD5	grey
LYVE1	black
LYZ	blue
MAGEL2	grey
MAN1A1	black
MAN1A2	turquoise
MAN1C1	turquoise
MAOB	black
MAP1LC3C	grey
MAP3K2	turquoise
MAP4K1	blue
MAP7D1	grey
MARCO	grey
MBNL3	turquoise
SLC25A53	turquoise

MCOLN2	grey
MED13	turquoise
MED13L	turquoise
MEF2B	grey
MEF2C	black
MEI1	blue
MEOX2	black
MET	black
MFAP3	turquoise
MFAP4	black
MFNG	blue
MGAT4A	turquoise
MGAT5	turquoise
MZB1	grey
MGP	black
MICAL2	turquoise
MICB	blue
MIR155HG	blue
MITF	black
MLPH	grey
MMP12	blue
MMP1	grey
MMP14	black
MMP16	black
MMP2	black
MMP25	grey
MMP3	grey
MMP9	blue
MMRN1	black
MMRN2	black
MNDA	blue
MPEG1	blue
MPL	turquoise
MPP1	grey
MR1	black
MRC1	blue
MRGPRF	black
MRO	grey
MRPL27	turquoise
MRPL55	turquoise
MRPS12	grey
MRPS21	turquoise
MRVI1	black
MS4A14	blue

MS4A1	blue
MS4A2	black
MS4A4A	blue
MS4A6A	blue
MS4A7	blue
MSR1	blue
MSRB3	black
MVP	blue
MX1	greenyellow
MXD1	turquoise
MXRA8	black
MYCT1	black
MYEF2	turquoise
MYO1F	blue
MYO1G	grey
MYO7A	grey
MYO9A	turquoise
N4BP2	turquoise
N4BP2L1	blue
NAALADL1	black
NAIP	blue
NAP1L3	black
NAPSB	blue
NBEA	turquoise
NBEAL1	turquoise
NCCRP1	blue
NCF1	blue
NCF1B	blue
NCF1C	blue
NCF2	blue
NCF4	blue
NCKAP1L	blue
NCOA2	turquoise
NCR1	blue
NCR3	blue
NEGR1	black
NFAM1	blue
NFATC2	blue
NFKB2	grey
NFKBID	blue
NFKBIE	blue
NFYA	turquoise
NFYB	turquoise
NFYC	turquoise

NHLRC2	turquoise
NHSL2	black
NID2	black
NIPAL4	black
NKG7	blue
NLRC3	blue
NLRC4	blue
NLRC5	blue
NLRP12	grey
NLRP3	blue
NMI	blue
NOD2	blue
NOTCH4	black
NOVA2	black
NOX4	black
NR1H3	blue
NR5A2	grey
NRP1	turquoise
NRXN3	grey
NT5E	black
NTM	grey
NTNG2	grey
NTRK1	black
OAS1	greenyellow
OAS2	greenyellow
OAS3	greenyellow
OASL	greenyellow
TENM3	black
OGFRL1	turquoise
OGN	black
OLFML1	black
OLFML2B	black
OLFML3	black
OLR1	blue
OMD	black
OSCAR	blue
OSM	blue
OSMR	turquoise
OTOA	grey
OVOL1	grey
P2RX1	black
P2RX4	grey
P2RX5	grey
P2RX7	blue

P2RY10	blue
P2RY11	grey
P2RY12	blue
P2RY13	blue
P2RY14	blue
P2RY8	blue
P4HA3	grey
PABPC5	turquoise
PADI2	blue
PAFAH1B2	turquoise
PAG1	turquoise
PAK5	grey
PALM2-AKAP2	black
PARM1	black
PARP12	grey
PARP14	greenyellow
PARP15	blue
PARP9	greenyellow
PARVG	blue
PATL2	blue
PATZ1	turquoise
PBX4	blue
PCDH12	black
PCDH17	blue
PCDH18	black
PCDHGA12	black
PCOLCE	black
PCYOX1L	grey
PDCD1	blue
PDCD1LG2	blue
PDE1A	grey
PDE1B	black
PDE3A	black
PDE3B	turquoise
PDE4B	turquoise
PDE6G	blue
PDGFA	grey
PDGFB	black
PDGFC	black
PDGFRA	black
PDGFRB	black
PDGFRL	grey
PDIA2	grey
PDIA3	turquoise

PDZK1IP1	grey
PDZRN3	turquoise
PECAM1	black
PEG3	turquoise
PFDN2	grey
PGLYRP4	grey
PGM5	black
PHACTR1	blue
PIK3AP1	turquoise
PIK3CG	blue
PIK3R5	blue
PIK3R6	blue
PILRA	blue
PIM2	blue
PIP4K2A	blue
PKD2L1	grey
PKHD1L1	grey
PKIB	grey
PKP3	grey
PLA1A	grey
PLA2G2D	blue
PLA2G4E	grey
PLA2G7	turquoise
PLAC9	black
PLCB2	blue
PLCB4	turquoise
PLCL1	black
PLCL2	blue
PLD4	blue
PLEK2	grey
PLEK	blue
PLEKHM3	turquoise
PLEKHN1	grey
PLEKHO1	black
PLEKHO2	blue
PLIN3	turquoise
PLVAP	black
PLXDC1	black
PLXNA4	grey
PLXNC1	blue
PLXND1	black
PML	grey
PMP22	black
PNMA2	black

PNOC	blue
PODN	black
POSTN	black
POU2AF1	blue
POU2F2	blue
PLPP3	black
PLPP4	grey
PLPP7	black
PPBP	grey
PPFIA2	grey
PPL	turquoise
PPM1H	turquoise
PPM1M	blue
PPP1R13L	grey
PPP1R16B	blue
PPP1R9A	turquoise
PRAM1	blue
PRELP	black
PREX1	blue
PREX2	black
PRF1	blue
PRG2	grey
PRKAR2A	turquoise
PRKAR2B	grey
PRKCB	black
PRKCQ	blue
PRKG1	black
PRLR	grey
PROCR	black
PROM1	blue
PRRX1	black
PRSS27	grey
PRTG	grey
PSAP	turquoise
PSMB10	blue
PSMB8	blue
PSMB9	blue
PSMC1	turquoise
PSMC2	turquoise
PSMC3	turquoise
PSMC4	turquoise
PSMC5	turquoise
PSMC6	turquoise
PSMD10	turquoise

PSMD11	turquoise
PSMD13	grey
PSMD14	turquoise
PSMD1	turquoise
PSMD2	turquoise
PSMD3	turquoise
PSMD4	grey
PSMD5	turquoise
PSMD6	turquoise
PSMD7	turquoise
PSMD8	turquoise
PSME1	grey
PSME2	grey
PSME3	turquoise
PSTPIP1	blue
PTAFR	blue
PTCRA	grey
PTGDR	blue
PTGDS	black
PTGER2	black
PTGFR	grey
PTGIR	blue
PTGIS	black
PTH1R	black
PTPN22	blue
PTPN6	blue
PTPN7	blue
PTPRB	black
PTPRC	blue
PTPRJ	turquoise
PTPRM	grey
PTPRO	blue
CAVIN1	black
PUS10	turquoise
PVR	turquoise
PVRIG	grey
NECTIN2	grey
NECTIN3	turquoise
PYHIN1	blue
QPRT	black
RAB20	grey
RAB33A	blue
RAB37	blue
RAB39A	grey

RAB39B	grey
RAB42	blue
RAB8B	turquoise
RAD23B	turquoise
RAD54L2	turquoise
RAET1E	grey
RAET1G	grey
RAET1L	grey
RAI2	black
RAMP3	black
RAPGEF2	turquoise
RAPGEF6	turquoise
RARRES2	black
RASAL3	blue
RASGRF2	black
RASGRP2	black
RASGRP3	blue
RASGRP4	blue
RASL12	black
RASSF2	blue
RASSF3	turquoise
RASSF4	blue
RASSF5	blue
RASSF6	turquoise
RBM38	blue
RBP5	blue
RC3H2	turquoise
RCAN2	black
RCN3	black
RCSD1	blue
RDH12	grey
RECK	black
REL	turquoise
RELB	blue
RELN	grey
RENBP	blue
REST	turquoise
RFTN1	black
RFX5	turquoise
RFXANK	turquoise
RFXAP	turquoise
RGL1	black
RGL4	blue
RGPD1	grey

RGS13	grey
RGS1	blue
RGS18	blue
RGS5	black
RHOD	grey
RHOH	blue
RHOJ	black
RIF1	turquoise
RIMKLA	turquoise
RIN1	black
RIN3	black
CARMIL2	blue
RNASE1	black
RNASE2	blue
RNASE6	blue
RNASE7	grey
RNF125	turquoise
RNF166	blue
RNF180	turquoise
RNF222	grey
LAMTOR2	turquoise
ROBO4	black
ROCK2	turquoise
ROR1	black
RPS6KA4	grey
RRN3P2	blue
RSAD2	greenyellow
RTKN2	turquoise
RTN1	grey
RTP4	blue
RUFY4	blue
RUNX1T1	black
RUNX3	blue
S100A12	blue
S100A16	blue
S100A2	grey
S100A8	blue
S100A9	blue
S100B	black
S1PR1	black
S1PR4	blue
SALL2	turquoise
SAMD14	black
SAMD3	blue

SAMD9	greenyellow
SAMD9L	greenyellow
SAMHD1	blue
SAMSN1	blue
SARDH	grey
SASH3	blue
SBNO1	turquoise
SBSN	grey
SCARF1	black
SCARF2	black
SCEL	grey
SCML4	blue
SCN7A	black
SCUBE3	black
SDC2	black
SDCBP2	grey
SDR9C7	grey
SDS	blue
SDSL	grey
SEC24A	turquoise
SEC24D	turquoise
SECISBP2L	turquoise
SECTM1	blue
SELENBP1	grey
SELL	blue
SELP	black
SELPLG	blue
SERINC5	turquoise
SERPINA1	blue
SERPINE1	black
SERPINF1	black
SERPING1	black
SFMBT2	turquoise
SFN	grey
SFRP2	black
SFTPB	grey
SGCD	grey
SGIP1	black
POMK	turquoise
PEAK1	black
SH2B3	blue
SH2D1A	blue
SH2D2A	blue
SH2D3C	black

SH2D5	grey
SHE	black
SEM1	turquoise
SIGLEC10	blue
SIGLEC11	blue
SIGLEC12	grey
SIGLEC14	blue
SIGLEC1	blue
SIGLEC5	blue
SIGLEC6	black
SIGLEC7	blue
SIGLEC8	blue
SIGLEC9	blue
SIGLEC17P	black
SIRPB1	blue
SIRPB2	blue
SIRPG	blue
SIT1	blue
SKAP1	blue
SLA2	blue
SLA	blue
SLAMF1	blue
SLAMF6	blue
SLAMF7	blue
SLAMF8	blue
SLC11A1	grey
SLC12A3	grey
SLC15A3	blue
SLC17A9	blue
SLC18A2	grey
SLC1A7	black
SLC24A4	grey
SLC25A45	grey
SLC29A3	grey
SLC2A5	grey
SLC34A2	blue
SLC39A2	grey
SLC45A3	grey
SLC6A12	grey
SLC7A7	blue
SLC8A1	black
SLCO2B1	blue
SLCO5A1	grey
SLIT2	black

SLIT3	black
SLURP1	grey
SMAP2	turquoise
SMPDL3B	grey
SNAI3	blue
SNED1	turquoise
SNRPF	grey
SNTB1	black
SNX20	blue
SOD3	grey
SON	turquoise
SOX17	black
SOX5	black
SP100	greenyellow
SP110	greenyellow
SP140	blue
SPAG4	grey
SPARC	black
SPARCL1	black
SPATA13	turquoise
SPI1	blue
SPIB	blue
SPN	blue
SPNS3	blue
SPOCK2	blue
SPON1	black
SPRY1	black
SRGN	blue
SSC5D	black
SSTR3	blue
ST3GAL2	black
ST3GAL5	blue
ST3GAL6	grey
ST6GAL1	turquoise
ST6GALNAC3	black
ST8SIA4	blue
STAB1	blue
STAC3	grey
STAP1	grey
STARD13	turquoise
STARD8	black
STAT1	greenyellow
STAT4	blue
STAT5A	blue

STK17B	turquoise
STK33	black
STRN	turquoise
STXBP6	grey
SUCNR1	grey
SULF1	black
SULT1C2	grey
SULT1C4	black
SULT2B1	grey
SUSD3	blue
SVOPL	grey
SYNE1	black
SYT11	black
SYTL3	grey
TAGAP	blue
TAOK1	turquoise
TAP1	blue
TAP2	blue
TAPBP	blue
TAPBPL	grey
TARP	grey
TBC1D10C	blue
TBCEL	turquoise
TBX21	blue
TBXA2R	black
TBXAS1	grey
TCEAL7	black
TCIRG1	grey
TCL1A	blue
TCN2	black
TEK	black
TESC	grey
TFEC	blue
TGFB1	black
TGFB2	black
TGFB3	black
TGFBI	black
TGFBR1	turquoise
TGFBR2	black
TGFBRAP1	turquoise
TGM1	grey
TGM2	blue
THBS1	black
THBS2	black

THEMIS	blue
THPO	grey
THSD7A	turquoise
THY1	black
TIE1	black
TIFAB	blue
TIGIT	blue
TIMD4	grey
PAM16	grey
TIMM50	grey
TIMP2	black
TIMP3	black
TLR10	blue
TLR1	blue
TLR4	black
TLR5	turquoise
TLR7	blue
TLR8	blue
TLR9	grey
TM4SF18	grey
TM6SF1	blue
DCSTAMP	grey
TMC8	blue
TMEM106A	black
TMEM119	black
TMEM140	greenyellow
IGFLR1	grey
TMEM150B	blue
TMEM156	blue
TMEM170B	turquoise
TMEM176A	blue
TMEM176B	blue
TMEM200A	black
TMEM204	grey
TMEM229B	grey
TMEM233	black
TMEM26	grey
TMEM47	black
TMEM79	grey
SYNDIG1	black
TMIGD2	blue
TNF	blue
TNFAIP6	grey
TNFAIP8L2	blue

TNFRSF10A	turquoise
TNFRSF10B	turquoise
TNFRSF10C	grey
TNFRSF10D	grey
TNFRSF11A	turquoise
TNFRSF11B	turquoise
TNFRSF12A	blue
TNFRSF13B	blue
TNFRSF13C	blue
TNFRSF14	grey
TNFRSF17	blue
TNFRSF18	grey
TNFRSF1A	grey
TNFRSF1B	blue
TNFRSF25	blue
TNFRSF4	blue
TNFRSF8	blue
TNFRSF9	blue
TNFSF10	grey
TNFSF11	blue
TNFSF12	black
TNFSF12-TNFSF13	grey
TNFSF13	turquoise
TNFSF13B	blue
TNFSF14	blue
TNFSF15	blue
TNFSF18	grey
TNFSF4	turquoise
TNFSF8	blue
TNFSF9	grey
TNIK	black
TNIP3	blue
TNN	grey
TNNT2	grey
TNS3	turquoise
TOX	grey
TPK1	grey
TPSAB1	black
TPSB2	black
TPSD1	grey
TPSG1	black
TRAF1	blue
TRAF3IP3	blue
TRANK1	greenyellow

TRAT1	blue
TREM1	blue
TREM2	blue
TREML1	blue
TRIM21	greenyellow
TRIM22	blue
TRIM61	black
TRPC4AP	turquoise
TRPV2	blue
TRPV3	grey
TSHR	grey
TSHZ3	black
TSLP	black
TSPAN11	black
TSPAN32	blue
TSPAN4	black
TTBK2	turquoise
TTC16	grey
TTC21B	turquoise
TTC24	blue
TTC37	turquoise
TUBA4A	grey
TUBB6	black
TXK	blue
NME8	blue
TYMP	blue
TYROBP	blue
UBA7	blue
UBASH3A	blue
UBD	blue
UBE2L6	blue
UBR1	turquoise
UBXN11	grey
UBXN1	turquoise
UCP2	blue
UHMK1	turquoise
ULBP1	grey
ULBP2	grey
ULBP3	turquoise
UNC13D	blue
UNC5C	black
UNC93B1	grey
USHBP1	black
USP12	turquoise

USP51	turquoise
UTS2	grey
VAMP5	black
VASH1	blue
VAV1	blue
VCAM1	blue
VCAN	black
VEGFA	grey
VEGFB	grey
VEGFC	grey
VENTX	blue
VGLL3	grey
VIM	black
VMO1	blue
VNN2	blue
VPREB3	blue
VPS37D	grey
VSIG4	blue
VWF	black
WARS	blue
WAS	blue
WDFY4	blue
WFDC12	grey
WIPF1	blue
WNT2	black
XAF1	greenyellow
XCL1	blue
XCL2	blue
XCR1	blue
XKR8	grey
XPNPEP2	black
ZAP70	blue
ZBP1	blue
ZBTB10	turquoise
ZBTB32	grey
ZC3H12D	blue
ZCCHC24	black
ZDHHC20	turquoise
ZEB1	black
ZEB2	black
ZFPM2	black
ZKSCAN1	turquoise
ZMYND15	blue
ZNF185	black

ZKSCAN8	turquoise
ZNF215	grey
ZNF366	black
ZNF423	black
ZNF469	black
ZNF521	black
ZNF620	turquoise
ZNF660	black
ZNF671	grey
ZNF683	blue
ZNF804A	black
ZNF80	blue
ZNF827	turquoise
ZNF831	blue
ZNF835	grey
