# Supplementary figures and images for: Development of a Novel Immune Subtyping System Expanded with Immune Landscape and an 11-Gene Signature for Predicting Prostate Cancer Survival
Source: J Oncol. 2022 Feb 16;2022:1183173. doi: 10.1155/2022/1183173 (PMC8866019; doi:10.1155/2022/1183173)

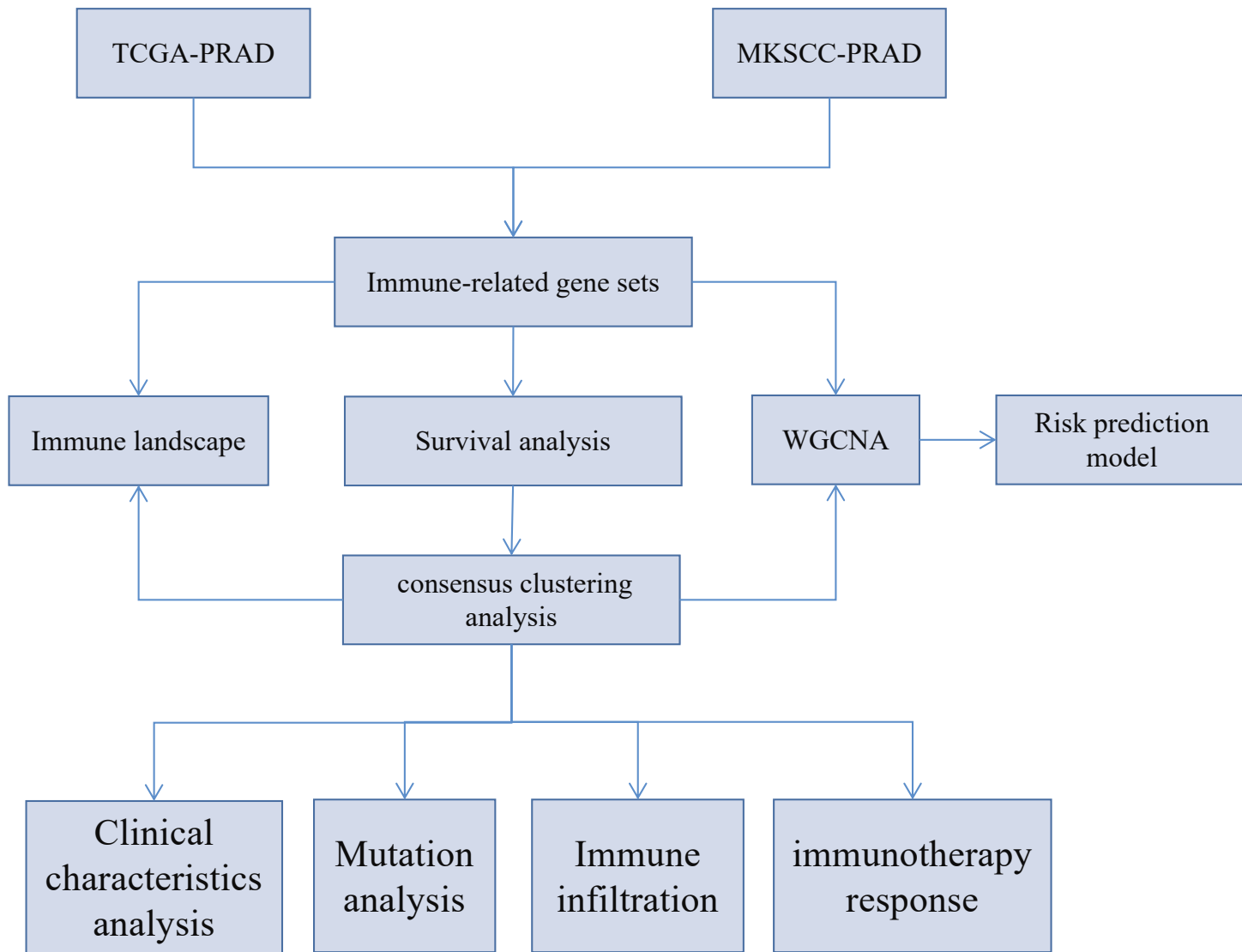

Supplement: Supplementary Materials — Supplementary Figure S1. Work flow chart. Supplementary Figure S2. Expression and interaction analysis of 11 genes. (A) Differential expression of 11 genes in cancer and adjacent tissues. (B) Correlation between 11 genes and immune infiltrating cells. (C) Correlation analysis between the expression of 11 genes and immune checkpoint genes. (D) Interaction between 11 genes. Supplementary Table S1. Immune related genes list. Supplementary Table S2. Clinical information of TCGA-PRAD dataset. Supplementary Table S3. Clinical information of MKSCC-PRAD dataset. Supplementary Table S4. Information of co-expression module corresponding to each gene. [file 1183173.f1.zip › 1183173.f1/Supplementary Figure S1.pdf]

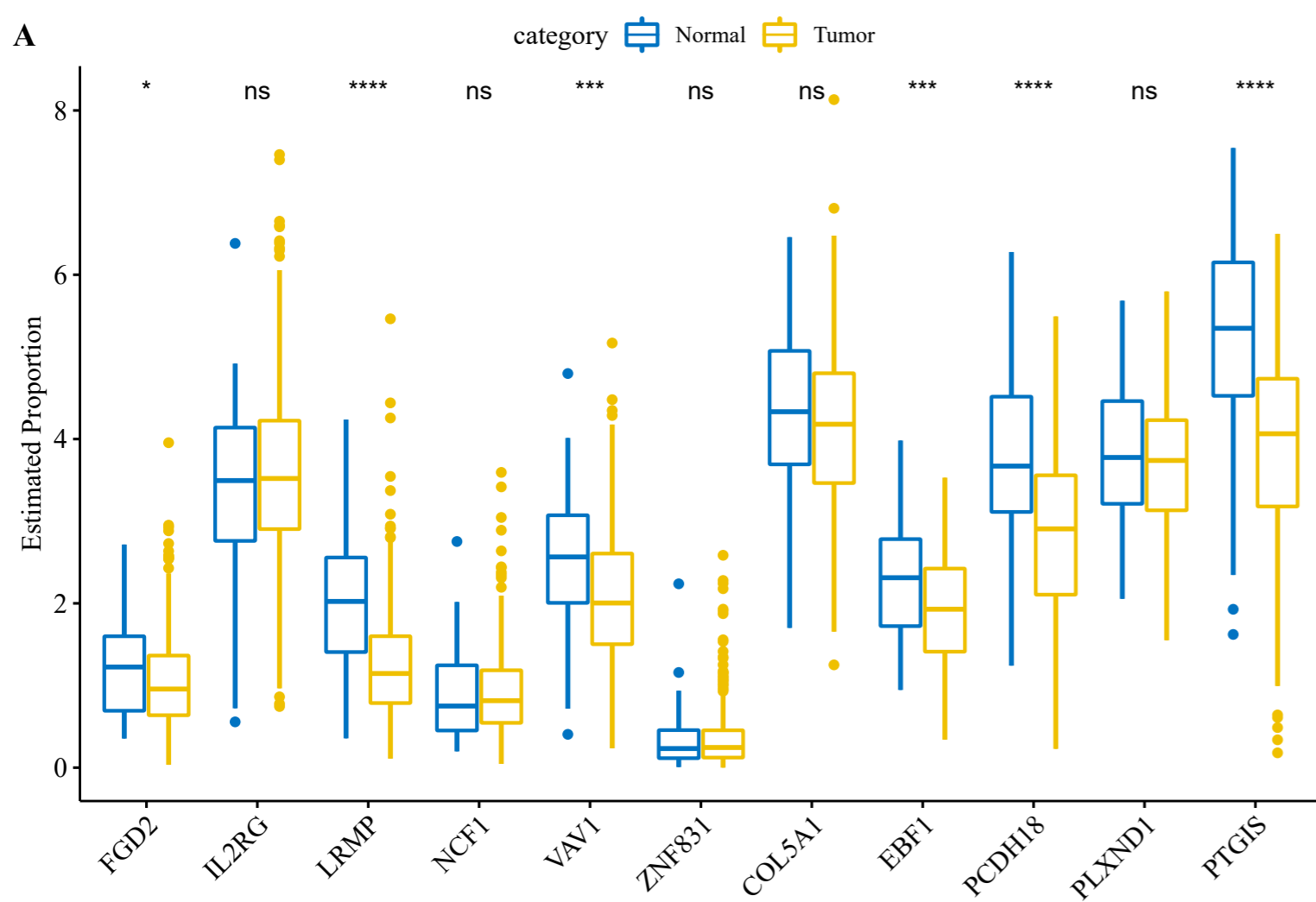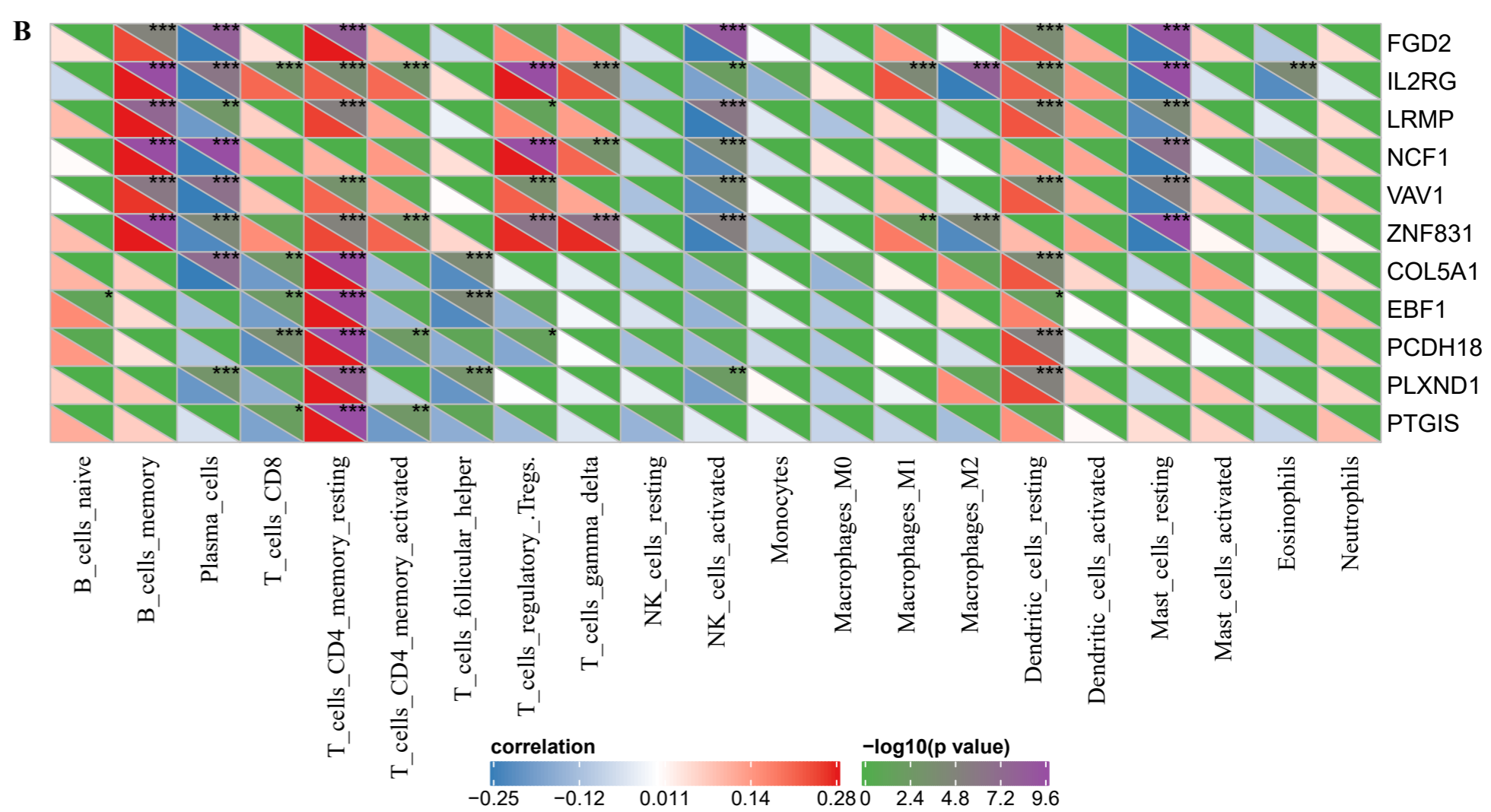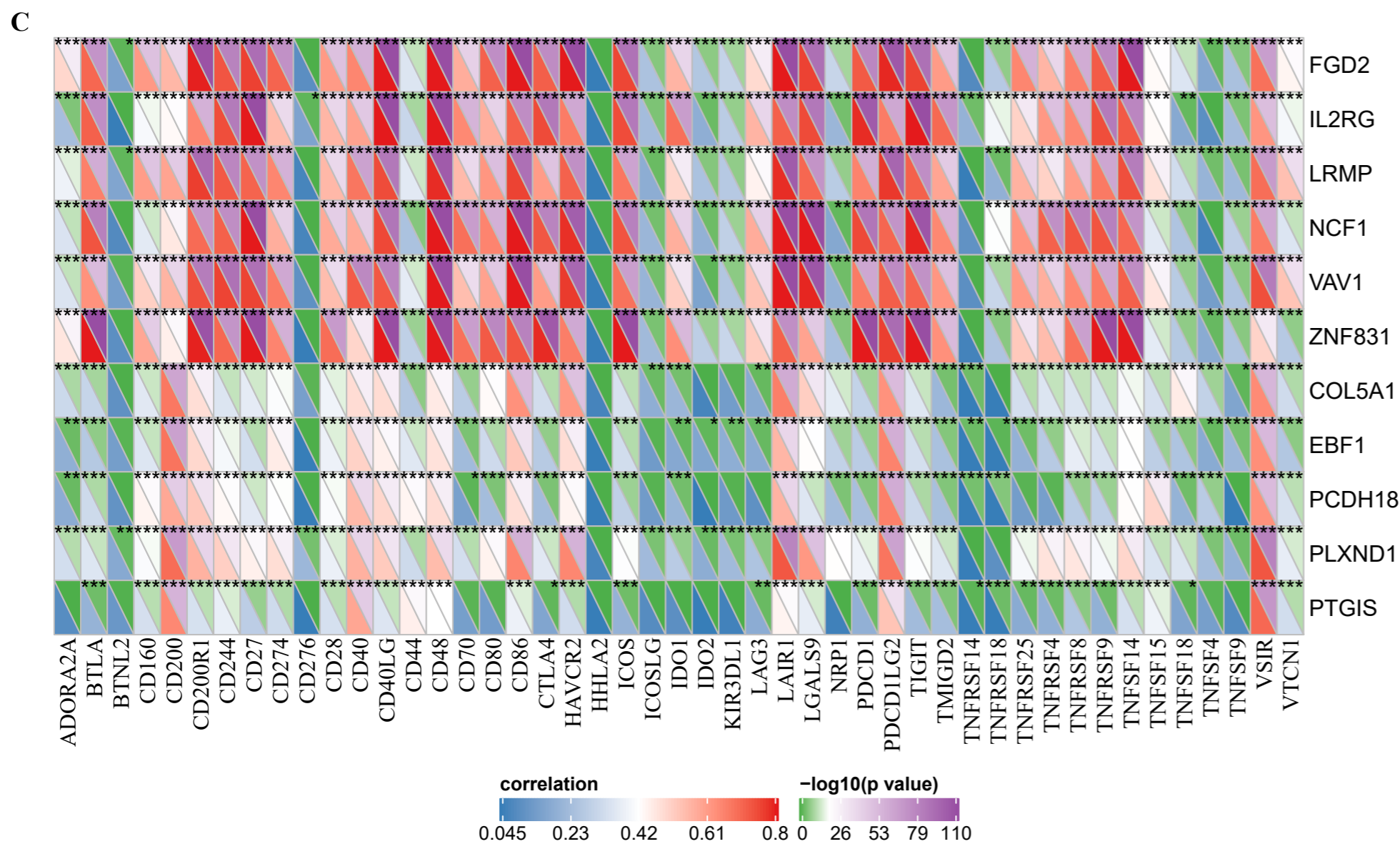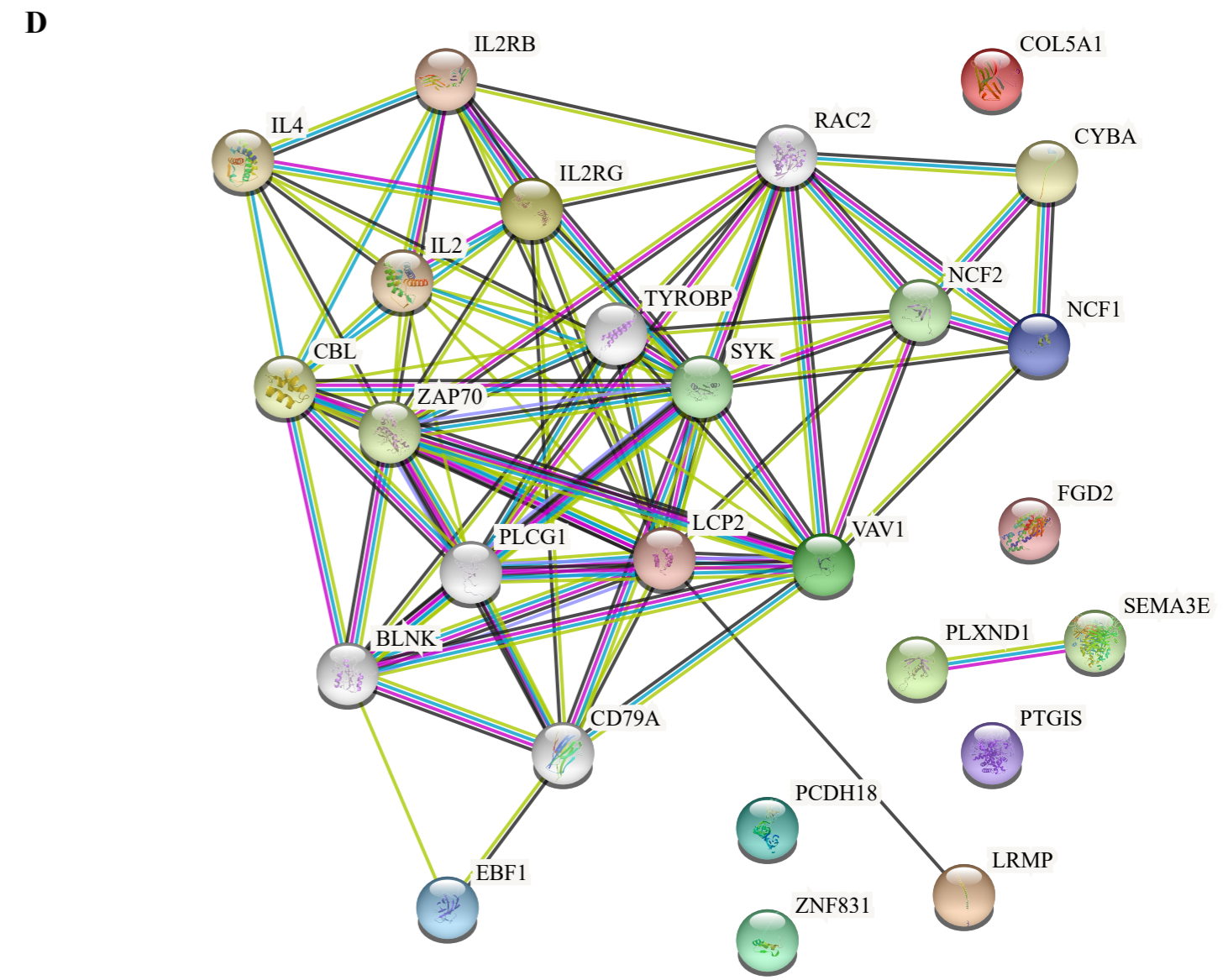

Supplement: Supplementary Materials — Supplementary Figure S1. Work flow chart. Supplementary Figure S2. Expression and interaction analysis of 11 genes. (A) Differential expression of 11 genes in cancer and adjacent tissues. (B) Correlation between 11 genes and immune infiltrating cells. (C) Correlation analysis between the expression of 11 genes and immune checkpoint genes. (D) Interaction between 11 genes. Supplementary Table S1. Immune related genes list. Supplementary Table S2. Clinical information of TCGA-PRAD dataset. Supplementary Table S3. Clinical information of MKSCC-PRAD dataset. Supplementary Table S4. Information of co-expression module corresponding to each gene. [file 1183173.f1.zip › 1183173.f1/Supplementary Figure S2.pdf]
